# Increased Drp1 promotes autophagy and ESCC progression by mtDNA stress mediated cGAS-STING pathway

**DOI:** 10.1186/s13046-022-02262-z

**Published:** 2022-02-24

**Authors:** Yujia Li, Hui Chen, Qi Yang, Lixin Wan, Jing Zhao, Yuanyuan Wu, Jiaxin Wang, Yating Yang, Menglan Niu, Hongliang Liu, Junqi Liu, Hushan Yang, Shaogui Wan, Yanming Wang, Dengke Bao

**Affiliations:** 1 Laboratory of Cancer Biomarkers and Liquid Biopsy, School of Pharmacy, Henan University, Kaifeng, 475004 Henan China; 2 School of Life Sciences, Henan University, Kaifeng, 475004 Henan China; 3 Nanyang Central Hospital, Henan University, Nanyang, 473000 Henan China; 4 Department of Radiation Oncology, the First Affiliated Hospital of Zhengzhou University, Zhengzhou, 450052 Henan China; 5 Department of Medical Oncology, Sidney Kimmel Cancer Center, Thomas Jefferson University, Philadelphia, PA 19107 USA; 6 Center for Molecular Pathology, First Affiliated Hospital, Gannan Medical University, Ganzhou, 341000 Jiangxi China

**Keywords:** Mitochondrial DNA stress, Autophagy, cGAS-STING signaling pathway, Drp1, Esophageal Squamous Cell Carcinoma

## Abstract

**Background:**

Mitochondrial dynamics homeostasis is important for cell metabolism, growth, proliferation, and immune responses. The critical GTPase for mitochondrial fission, Drp1 is frequently upregulated in many cancers and is closely implicated in tumorigenesis. However, the mechanism underling Drp1 to influence tumor progression is largely unknown, especially in esophageal squamous cell carcinoma (ESCC).

**Methods:**

Immunohistochemistry was used to examine Drp1 and LC3B expression in tissues of ESCC patients. Autophagic vesicles were investigated by transmission electron microscopy. Fluorescent LC3B puncta and mitochondrial nucleoid were observed by fluorescent and confocal microscopy. Mitochondrial function was evaluated by mitochondrial membrane potential, ROS and ATP levels. Xenograft tumor model was performed in BALB/c nude mice to analyze the role of Drp1 on ESCC progression.

**Results:**

We found that Drp1 high expression is correlated with poor overall survival of ESCC patients. Drp1 overexpression promotes cell proliferation and xenograft ESCC tumor growth by triggering autophagy. Furthermore, we demonstrated that Drp1 overexpression disturbs mitochondrial function and subsequent induces mitochondrial DNA (mtDNA) released into the cytosol thereby inducing cytosolic mtDNA stress. Mechanistically, cytosolic mtDNA activates the cGAS-STING pathway and facilitates autophagy, which promotes ESCC cancer growth. Moreover, mtDNA digestion with DNase I and autophagy inhibition with chloroquine attenuates the cGAS-STING pathway activation and ESCC cancer growth.

**Conclusions:**

Our finding reveals that Drp1 overexpression induces mitochondrial dysfunction and cytosolic mtDNA stress, which subsequently activates the cGAS-STING pathway, triggers autophagy and promotes ESCC progression.

**Supplementary Information:**

The online version contains supplementary material available at 10.1186/s13046-022-02262-z.

## Background

Mitochondria are vital intracellular organelles involved in cellular metabolism, ATP generation and more recently, innate immunity [1, 2]. Accumulating evidence suggests that mitochondrial mass and dynamics play a central and multi-functional role in tumor progression by modulating cell cycle, redox homeostasis, metabolism and immune signals [3, 4]. Previous studies have demonstrated that the critical GTPase for mitochondrial dynamics, Drp1 is frequently upregulated in lung cancer and hepatocellular carcinoma (HCC), which is closely implicated in tumorigenesis [5, 6]. However, the underling mechanism by Drp1 influence tumor progression is largely unknown, especially in esophageal squamous cell carcinoma (ESCC).

Recently, damage-associated molecular patterns (DAMPs) from mitochondria were implicated in various chronic inflammatory diseases such as trauma, autoimmune disorders, ischemic heart disease and cancer [7, 8]. As a major mitochondrial DAMPs, mitochondrial DNA (mtDNA) plays an important role in tumor progression and metastasis [9, 10]. Using heterozygote TFAM (mitochondrial transcription factor A) knockout mice, it was demonstrated that TFAM deficiency causes markedly induces mtDNA instability and cytoplasm release, which is recognized as cytosolic mtDNA stress [2]. Further studies showed that during hypoxia, nuclear HMGB1 translocate to the cytoplasm and binds to mtDNA, which then activates TLR9 signaling and tumor proliferation in hepatocellular carcinoma (HCC) [11]. We previously found that increased mitochondrial fission through upregulated Drp1 significantly induced cytosolic mtDNA stress and subsequent CCL2 secretion in HCC cells, leading to infiltration of tumor-associated macrophages into HCC tissues and tumor progression [12]. However, whether mtDNA stress is involved in esophageal squamous cell carcinoma (ESCC) progression remains poorly understood.

cGAS (cyclic GMP-AMP synthase) is a cytosolic DNA sensor, which activates STING (stimulator of interferon genes) through the second messenger molecule cGAMP (2′3’-cGMP-AMP) to induce innate immunity [13, 14]. Recent studies found the cGAS-STING signaling cascade is involved in anticancer immune responses and cancer immune escape, which plays dichotomous effects on tumor development [15–18]. The cGAS-STING pathway activation by tumor-derived DNA can induce cellular senescence, pro-inflammatory cytokine expression, and immuno-surveillance to suppress tumorigenesis by promoting the release of type I interferons (IFNs) [15, 16]. Further, the STING activation by cytosolic DNA is involved in mediating innate immune recognition of immunogenic tumors [19]. However, prolonged type I IFNs signaling induced by the cGAS-STING pathway can cause immune dysfunction and promote tumor development [17, 20]. Moreover, STING signaling also facilitates tumor growth, chemoresistance and metastasis in breast cancer and lung cancer mainly by creating an immune suppressive tumor microenvironment [21–25]. Cytosolic mtDNA, which leaks under mitochondrial stress, can also trigger the cGAS-STING pathway and induce the production of inflammatory cytokines and type I IFNs [2, 26, 27]. However, it is unclear if mtDNA stress activates the cGAS-STING pathway in the context of ESCC progression.

Autophagy is evolutionarily conserved cellular process wherein cellular components such as macromolecules and organelles degrade through sequentially formed autophagic vesicles including phagophores, autophagosomes and autolysosomes [28]. The formation of autophagic vesicles and the recruitment of cellular components into autophagic vesicles are tightly regulated by a series of autophagy factors, including LC3B and p62. It had been reported that autophagy is also a cellular process adapted by cancer cells to cope with various adverse conditions, such as oxidative stress, ER stress, mitochondria stress and starvation [29]. Autophagy plays a tumor suppression or promotion role depending on cellular context and tumor microenvironment, and autophagy inhibitors have been tested in combination with other chemotherapy reagents in clinical trials [30–32].

In this study, we examined the role of Drp1 on ESCC progression in vitro and in vivo. Furthermore, we investigated whether the cGAS-STING pathway mediated autophagy is implicated in Drp1-induced ESCC progression. Our findings provide novel insight into the molecular mechanisms underlying mitochondrial dynamics and ESCC progression.

## Methods and materials

### Animals

Four-week-old female BALB/c nude mice were randomly divided into indicated groups, at least 7 each group. Xenograft nude mice model was established by subcutaneously injecting ESCC cells (5 × 10^6^ cells per mice) into the right flanks. Tumor size was measured weekly, and volume was calculated according to the following formula: volume = (Width^2^ × Length)/2. After injection 6 weeks, all mice were sacrificed and the tumors were harvested and weighted. The tumor tissue samples were evaluated by H&E staining and immunohistochemistry. Animal study was approved by the Institutional Animal Care and Use Committee of Henan University.

### Human tissue samples

A total of 61 paired ESCC tissue specimens from both tumor and adjacent normal tissues were obtained from untreated patients who underwent surgical treatment for ESCC at the Nanyang central Hospital of Henan University. The latest follow-up date was August 2017 and the clinical characteristics of all patients was distributed in Table S1. All samples were cut into two pieces. One piece was embedded in paraffin and processed for routine histopathological examination, while the other piece of tissue was frozen immediately in liquid nitrogen and stored at -80℃ for further studies. All patients were clinically and pathologically diagnosed to have ESCC. This study was approved by Ethics Committee of Henan University, and the informed consents were signed by all participants.

### Cell culture

Human ESCC cell lines KYSE-30, KYSE-70, KYSE-140, EC9706, KYSE-30 and EC9706 with Drp1 overexpression, KYSE-70 and KYSE-140 with Drp1 knockdown, and normal esophageal epithelial cell line Het-1A were routinely cultured in RPMI-1640 (10,040-CVR, Corning) medium supplemented with 10% fetal bovine serum (04–001-1Acs, BI). All cell lines were verified based on cell morphology and authenticated using short tandem repeat (STR) DNA testing.

### Knockdown and forced expression of target genes

For transfection, KYSE-30, KYSE-140, EC9706 and KYSE-70 cells were seeded in 6-well plates to 60% to 80% confluence. Then the vectors with or without target gene were respectively transfected into ESCC cells using the Lipofectamine 2000 reagent (11,668,019, Invitrogen) according to the manufacturer’s instructions. Stable transfectants were generated by limiting dilution after selection with G418 (10,131,027, Invitrogen) for 3 weeks. The recombinant DNA used in this study are listed in Table S2. All siRNAs were synthesized by GenePharma (Shanghai, China). The siRNA sequences of Drp1, TFAM, TBK1, STING, and cGAS are provided in Table S2. The siRNAs were transfected with Lipofectamine 2000 (11,668,019, Invitrogen) reagent according to the manufacturer’s protocol.

### Determination of mtDNA copies in cytosolic extracts

Cytosol fraction of ESCC cells were isolated using the cell mitochondria isolation kit (C3601, Beyotime) following the manufacturer’s instructions as previously described [12]. In brief, 1 × 10^7^ cells were incubated in 0.1 ml ice-cold mitochondrial lyses buffer for 10 min and homogenized with a Dounce homogenizer for 30 strokes. The homogenate was centrifuged at 600 × g for 10 min at 4 °C to remove nuclei and unbroken cells. The supernatant was collected and centrifuged again at 12,000 × g for 30 min at 4 °C for production of a supernatant corresponding to the cytosolic fraction. DNA of cytosolic fractions were isolated using TIANamp Genomic DNA Kit (DP304, TIANGEN) according to the manufacturer’s protocol. Cytosolic mtDNA was measured using the QX200™ Droplet Digital™ PCR System (QX200™, Bio-Rad Laboratories). Briefly, the 20 μl ddPCR reaction consisted of 2 × ddPCR Super Mix for probe 10 μl, 900 nM ND1-Forward and ND1-Reverse primers, 500 nM probe for ND1. The ddPCR reaction mixture and 70 μl of droplet generation oil for probe were placed into the DG8 cartridge (QX200™, Bio-Rad Laboratories) after equilibrating for 3 min at room temperature. After generating by droplet generator (QX200™, Bio-Rad Laboratories), the droplet emulsion was transferred to 96-well PCR plate (QX200™, Bio-Rad Laboratories) and subsequently heat-sealed using PX1™ PCR plate sealer (QX200™, Bio-Rad Laboratories) and PCR reaction was performed with a condition as follows: 95℃ for 10 min; 40 cycles of 95℃ for 30 s, 55℃ for 1 min; and a final step at 72℃ for 10 min. The absolute mtDNA concentration was analyzed by the QX200 droplet reader software, version 1.6.6.0320 (QX200™, Bio-Rad Laboratories) after PCR amplification.

### qRT-PCR, Western blot, IHC, and H&E staining.

Total RNA extraction, complementary DNA synthesis, and qRT-PCR were performed as previously described [12]. Primer sequences of qRT-PCR were provided in Table S2. ESCC tissues and cell lines were processed for western blot and IHC as previously described [12]. The band intensity on the western blots was quantified using Quantity One software (Hercules, Bio-Rad). Antibodies were shown in Table S3. The fold change between tumor and adjacent nontumor tissues were log_2_-transformed for further analysis.

### Autophagic vesicles imaging by transmission electron microscopy and mitochondrial nucleoid imaging by confocal microscopy

Cells were fixed with 4% glutaraldehyde and post fixed with 1% OsO_4_ in 0.1 M cacodylate buffer containing 0.1% CaCl_2_ for 2 h at 4 °C. The samples were then stained with 1% uranyl acetate, dehydrated in increasing concentrations of ethanol and embedded in araldite. Thin sections were stained with uranylacetate and lead citrate and analyzed with an electron microscope (Tecnai G2, FEI), at 11,500 magnifications.

ESCC cells were first with washed with HBSS and incubated with MitoTracker Red FM (M7512, Invivogen) for 0.5 h, and then incubated with Picogreen (P2023, UE) after another washing with HBSS for 1.5 h. Then, the distribution of mitochondrial nucleoids in living ESCC cells mitochondrial morphology was immediately monitored using Nikon N-SIM Structured Illumination microscope (Nikon/A1 + N-SIM, Nikon). For morphometric analysis, Image J software (NIH, Bethesda, MD) was used to measure the length of mitochondria.

### Cell viability and proliferation assays

Cell viability was determined by the MTS assay (G3580, Promega Corporation) according to the manufacturer’s instructions. Briefly, 2000 cells/well were plated in each well of a 96-well culture plate. After 1, 2, 3, 4, 5 days, cell viability was measured by addition of 20 μl of MTS solution and incubation for another 3 h. The absorbance of optical density (OD) was determined by PerkinElmer EnSpire microplate reader with a 490 nm wavelength. Each group were performed in triplicate. Cell’s proliferation was determined by colony formation assay and ethynyl deoxyuridine (EdU) incorporation assay (C10310-1, Ribbio). ESCC cells were inculcated into 6-well plate with 1000 cells/well and cultured for two weeks at 5% CO_2_, 37℃. Then, cells were fixed with 4% paraformaldehyde for 15 min, washed three times with PBS and incubated with 0.5% crystal violet solution for another 5 min. The colonies were counted and assays from three independent experiments. EdU incorporation assay was used to evaluate the proliferation of cells according to the manufacturer’s instructions (C10310-1, Ribbio). Then results were analyzed with a fluorescence microscope (U-LH100HG, Olympus Corporation).

### RNA sequencing

Total RNA of ESCC cells as indicated were extraction as previously described [12]. The integrity of RNA from ESCC cells were examined with Bioanalyzer 2100 (Agilent, Santa Clara, CA), and RNA samples with RIN > 7 were subjected to library construction and sequenced on Illumina novaseq by Genewiz Suzhou, In. Differentially expressed of mRNAs were analyzed as previous reported [33].

### Detection of reactive oxygen species, mitochondrial membrane potential and ATP measurement

Cellular reactive oxygen species (ROS) levels were detected by the fluorescent probe DCFH-DA (S0033S, Beyotime Biotechnology) following the manufacturer’s protocols. Briefly, ESCC cells suspension was incubated with 10 μM DCFH-DA, which diluted with serum free medium, at 37 °C for 20 min. The fluorescence was assessed by flow cytometry.

JC-1 dye (C2006, Beyotime Biotechnology) was used to detect the mitochondrial membrane potential. Cells were adjusted to a density of 1 × 10^5^/ml and stained with 5 mg/L JC-1 dye for 20 min at 37℃. After washing with the dye buffer three time, the cells were observed to a fluorescence microscope (U-LH100HG, Olympus Corporation) and detected fluorescence intensity ratio of JC-1 monomers to JC-1 aggregates (ratio of 529:590 nm emission intensity).

The total ATP were measured by plating 1 × 10^5^ cells in 6-well plates overnight, and then the cells were counted for ATP. The total ATP levels were determined using the Cell Titer-Glo Luminescent assay (G7570, Promega) according to the manufacture. The data were normalized to the number of cells.

### DNase I treatment

DNase I was delivered into cytosol using PULSin™ protein delivery reagent (501–01, Polyplus Transfection) following the manufacturer’s protocols. Firstly, 3 μg DNaseI was mixed with PULSin according to the manufacturer’s instructions. After washing three times using serum-free RPMI-1640, the cells were incubated with DNase I/PULSin mixture for 4 h at 37℃. Then, removing the mixture and incubating in fresh complete medium for another 24 h. Finally, the cells were collected for further studies.

### Evaluation of fluorescent LC3B puncta

ESCC cells were first transiently transfected with pcDNA3.1-GFP-LC3B (B5500, GenePharma) and then stained with Hoechst 33,342 (H4047, UE). The LC3B fluorescent puncta were viewed using Nikon N-SIM Structured Illumination microscope (Nikon/A1 + N-SIM, Nikon). The average number of GFP-LC3B puncta per cell in 5 high-power fields (HPF, 400 ×) was calculated, which was identified as autophagy.

### Statistics and reproducibility

Unpaired Student’s t-tests (two-sided) were used for comparisons between two groups where appropriate. Error bars represent standard error of mean. For prognosis analysis, variables (the IHC score of Drp1, LC3B) were analyzed dichotomically. The Kaplan–Meier survival curve and log-rank test were used to distinguish subgroup patients who had different overall survival. People who performed lab work were blinded to patients’ clinical data and no blinding was done for all animal studies. For every figure, the statistical tests are justified as appropriate and the data meet the assumptions of the tests. All experiments were technically repeated at least three times. SPSS 20.0 software (IBM, Chicago, IL) was used for all statistical analyses and *p* < 0.05 was considered statistically significant.

## Results

### Drp1-mediated mitochondrial fission promoted survival of ESCC cells in vitro* and *in vivo

**Fig. 1:**
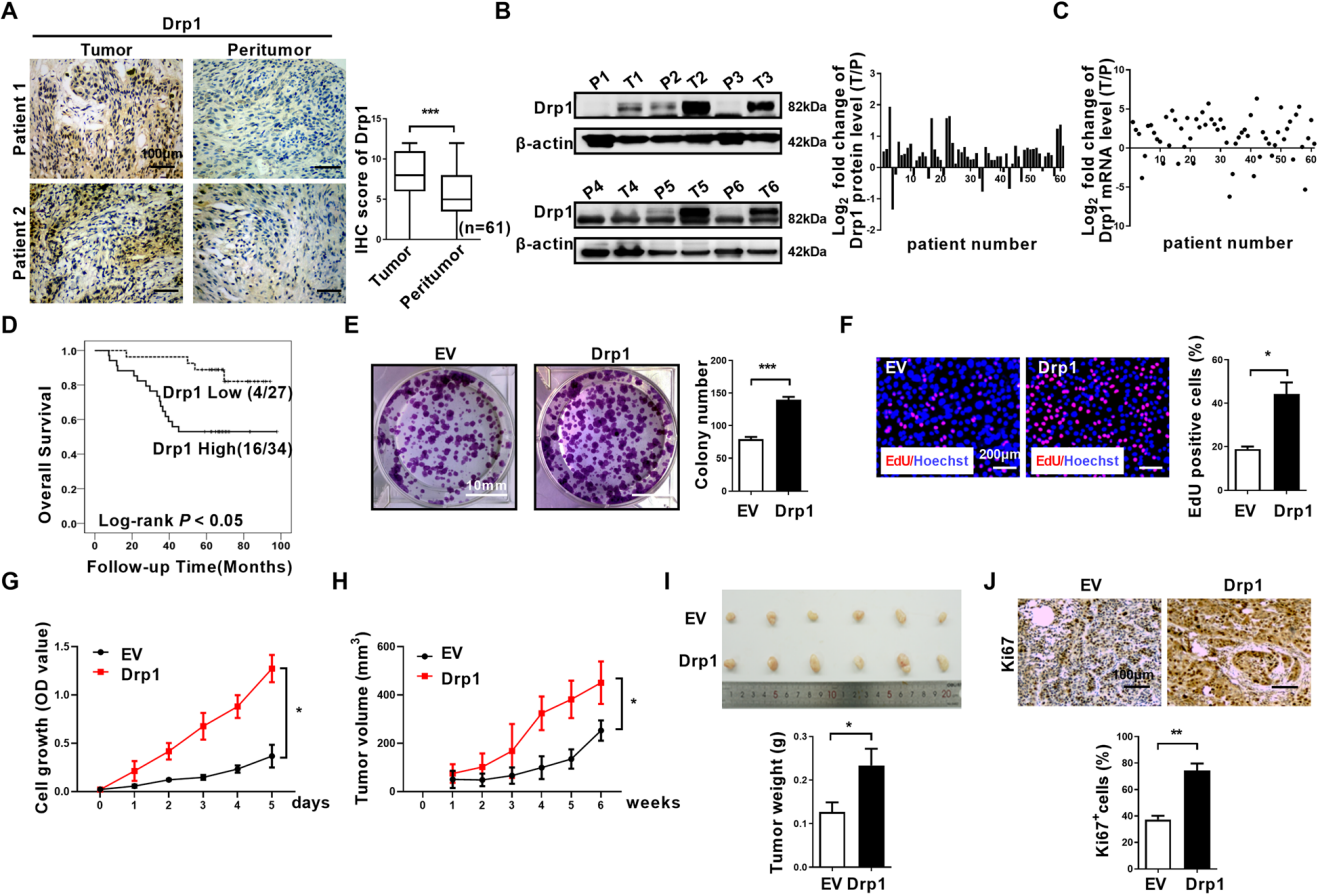
**Drp1-mediated mitochondrial fission promoted survival of ESCC cells in vitro and in vivo. (A)** Representative immunohistochemical (IHC) staining images of Drp1 and TFAM in paired ESCC tissues (*n* = 61). Scale bar: 100 μm. **(B and C)** Western blot and qRT-PCR analyses for the expression levels of Drp1 TFAM in paired tissues from ESCC patients. T, tumor; P, peritumor. The relative expression ratio of tumor to peritumor was log_2_-transformed for further analysis. **(D)**Kaplan–Meier curve analysis of overall survival in ESCC patients grouped by the expression of Drp1 in tumor tissues. Patients were grouped into high or low level using the median value of IHC scores. Death/total number of patients in each subgroup were presented. **(E)**Colony-forming potential were detected in ESCC cells with treatment as indicated. Scale bar: 10 mm. The data shown are the mean ± SEM from three separate experiments. **(F)** Cell proliferation was evaluated by EdU incorporation assay in KYSE-30 cells as indicated. Scale bar: 200 μm. **(G)** Cell viability of KYSE-30 ESCC cells with Drp1 overexpression were evaluated using the MTS cell proliferation assay. **(H and I)** Tumor growth curves and weight of subcutaneous xenograft tumor model developed from ESCC cells with Drp1 overexpression (*n* = 6). The tumor volumes were calculated according to the formula (L × W^2^)/2 and presented as mean ± SEM. **(J)** Representative immunohistochemical (IHC) staining images of Ki67 in xenograft tumors developed from KYSE-30 cells with Drp1 overexpression. Scale bar: 100 μm. The data shown are the mean ± SEM from three separate experiments. Drp1, expression vector encoding Drp1; EV, empty vector. *P* value from *t* tests. *, *P* < 0.05; **, *P* < 0.01; ***, *P* < 0.001

To investigate whether mitochondrial regulators Drp1 is involved in ESCC progression, we analyzed Drp1 expression in a cohort of 61 ESCC patients. Through IHC staining, we found that Drp1 expression was significantly upregulated in ESCC tumors in comparison to peritumor tissues (Fig. 1A). We further performed protein and mRNA analyses and found that tumors tend to express higher amounts of Drp1 than peritumor tissues (Fig. 1B and C). Moreover, patients with higher Drp1 expression had significantly shorter overall survival (Fig. 1D), suggesting that Drp1 plays pivotal roles in ESCC progression.

The above results prompted us to investigate the molecular functions of Drp1 in ESCC progression. We first investigated the expression of Drp1 in several ESCC cell lines and normal esophageal epithelial cell line Het-1A. We found that Drp1 expression was much higher in ESCC cells compared with Het-1A (Fig. S1A-B). In ESCC cell lines, the Drp1 expression was much higher in KYSE-140 and KYSE-70 cells compared with EC9706 and KYSE-30 cells (Fig. S1A-B). Next, we performed Drp1 overexpression with a vector in KYSE-30 and EC9706 ESCC cells which have relatively low Drp1 expression levels (Fig. S1A-F). We found that Drp1 overexpression increased the percentage of cells with fragmented mitochondria, with a decreased percentage of cells with elongated mitochondria (Fig. S1G-H). Next, we analyzed the potential roles of Drp1 on the growth of ESCC. Elevation of Drp1 expression in the KYSE-30 and EC9706 ESCC cell line significantly increased colony formation, EdU incorporation, and cell growth rate (Fig. 1E-G, and Fig. S2). The results were confirmed by a subcutaneous xenograft model in nude mice. The growth curve and tumor weight analyses found that in KYSE-30 cells derived xenograft tumor, elevation of Drp1 expression significantly increased tumor growth rate and tumor weight (Fig. 1H and I), and increased the percentage of Ki67^+^ cells in the xenograft tumors (Fig. 1J). These results indicate that Drp1 accelerates xenograft tumor growth of ESCC.

### Drp1 inhibitor or knockdown exhibits an anticancer effect on ESCC in vitro* and *in vivo

**Fig. 2:**
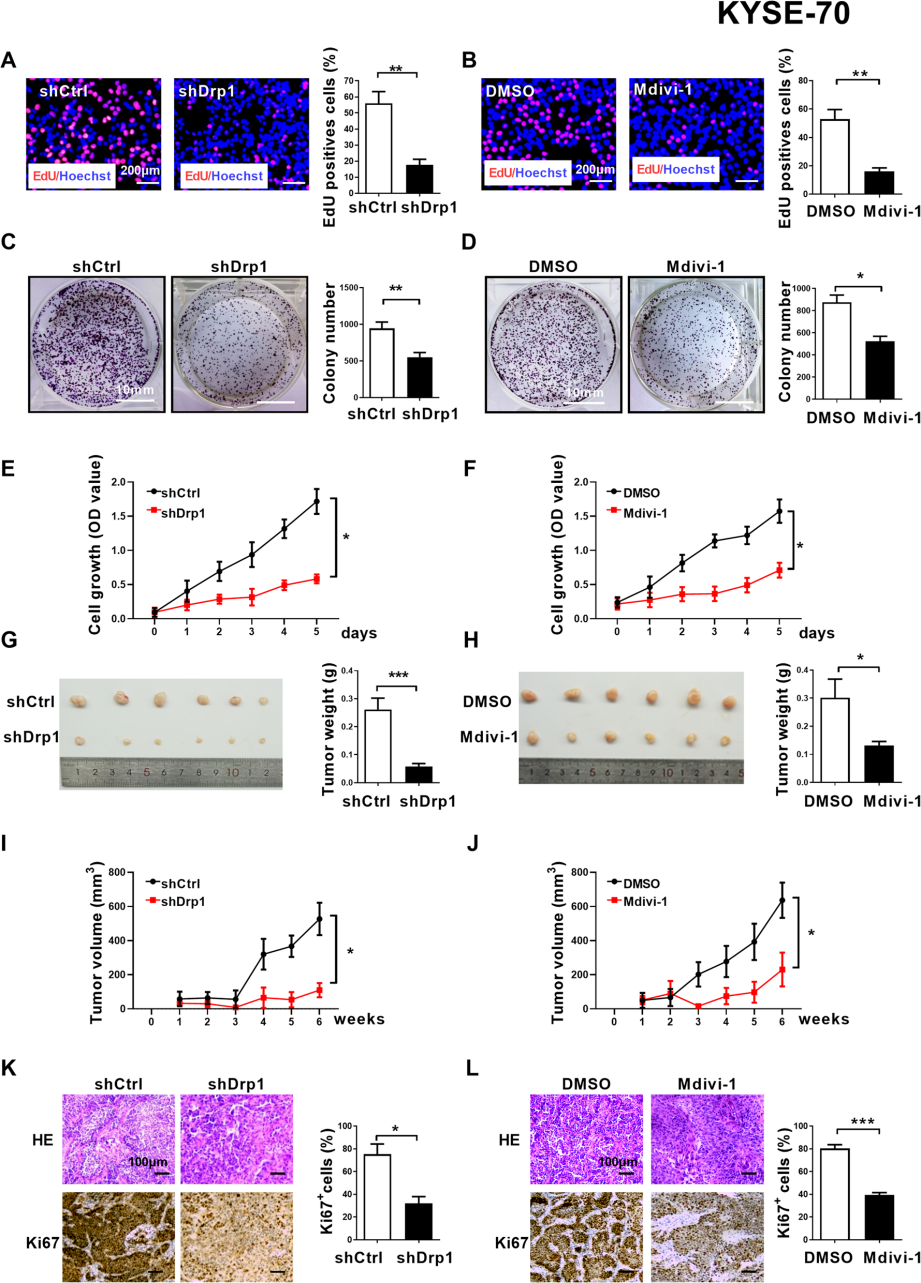
**Drp1 inhibitor or knockdown exhibits an anticancer effect on ESCC in vitro and in vivo. (A and B)** Cell proliferation was evaluated by EdU incorporation assay in ESCC cells with Drp1 knockdown or treatment with 50 μM Mdivi-1 for 12 h as indicated. Scale bar: 200 μm. **(C and D)** Colony-forming potential were detected in ESCC cells with Drp1 knockdown or treatment with 50 μM Mdivi-1 for 12 h as indicated. Scale bar: 10 mm. **(E and F)** Cell viability of ESCC cells with Drp1 knockdown or treatment with 50 μM Mdivi-1 for 12 h as indicated were evaluated using the MTS cell proliferation assay. **(G, H and I, J)** Tumor growth curves of subcutaneous xenograft tumor model developed from ESCC cells with Drp1 deficiency (*n* = 6) or treatment with Mdivi-1 (*n* = 6). Tumor size including tumor length (L) and width (W) was measured using vernier calipers every week from second week after transplantation. The tumor volumes were calculated according to the formula (L × W^2^)/2 and presented as mean ± SEM. Tumors from sacrificed mice were dissected fifth week after transplantation and were also shown in lower panel. **(K and L)** Representative immunohistochemical (IHC) staining images of Ki67 in xenograft tumors developed from KYSE-70 cells with Drp1 deficiency or treatment with Mdivi-1. Scale bar: 100 μm. The data shown are the mean ± SEM from three independent experiments. *, *P* < 0.05; **, *P* < 0.01; ***, *P* < 0.001. shDrp1, shRNA expression vector against Drp1; shCtrl, control shRNA

To explore the potential roles of Drp1 on ESCC cells survival, Drp1 expression was knockdown by shRNA or blockage by Drp1 selective inhibitor Mdivi-1 in KYSE-70 and KYSE-140 cells which have relatively high Drp1 expression levels (Fig. S1A-B, S3A-B, and S4A-B). The percentage of cells with fragmented mitochondria significantly reduced and the percentage of cells with elongated mitochondria significantly increased with Drp1 knockdown or Mdivi-1 treatment (Fig. S3C-D, and S4C-D). Consistently, Drp1 knockdown or Mdivi-1 treatment were also can inhibit ESCC cells survival in vitro and in vivo*,* which were investigated by EdU incorporation assay, MTS assay and colony formation assay and xenograft nude mice model (Fig. 2, and Fig S5). These data further support that Drp1 maybe a critical factor for ESCC progression.

### Drp1 overexpression regulates autophagy in ESCC cells

**Fig. 3:**
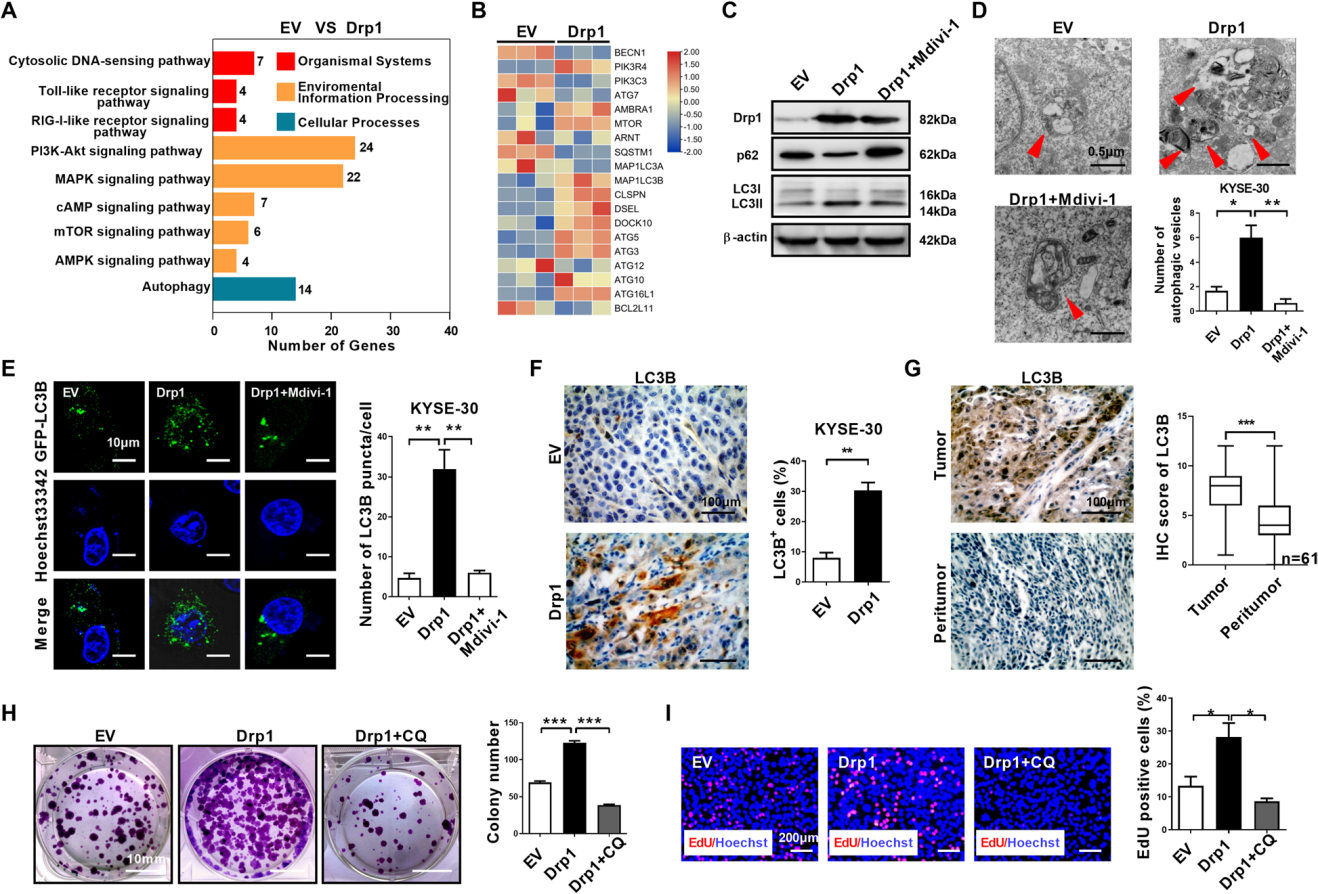
**Drp1 overexpression regulates autophagy in ESCC cells. (A)** RNA sequencing was performed on ESCC cells as indicated to screen differentially expressed mRNAs. Signaling pathway were plotted according to enriched gene ratio. **(B)** Heat map analysis of RNA sequencing for autophagy associated gene expression in ESCC cells as indicated. **(C)** Western blot analyses were performed in ESCC cells as indicated with antibodies specific for the proteins shown. **(D)** Representative transmission electron microscopy images of double-membrane autophagosomes in ESCC cells as indicated. Scale bar: 0.5 μm. **(E)** Representative images of fluorescent LC3B puncta (green) in ESCC cells as indicated. Scale bar: 10 μm. **(F)** Representative immunohistochemical (IHC) staining images of LC3B in xenograft tumors developed from KYSE-30 cells as indicated. Scale bar: 100 μm. **(F)** Colony-forming potential were detected in KYSE-30 cells as indicated. Scale bar: 10 mm. **(G)** Representative immunohistochemical (IHC) staining images of LC3B in paired ESCC tissues (*n* = 61). Scale bar: 100 μm. **(H and I)** Cell proliferation was evaluated by colony formation EdU incorporation assay in KYSE-30 cells as indicated. Scale bar: 200 μm. The data shown are the mean ± SEM from three separate experiments. Drp1, expression vector encoding Drp1; EV, empty vector. *P* value from *t* tests. *, *P* < 0.05; **, *P* < 0.01; ***, *P* < 0.001

To investigate the underlying mechanisms of Drp1 overexpression-mediated ESCC progression, transcriptome profiling was performed by RNA sequencing analysis in ESCC cells. We identified 316 upregulated genes and 564 downregulated genes after Drp1 overexpression (Table S4). KEGG pathway analysis revealed that the differentially expressed genes were significantly enriched in the autophagy and mTOR signaling pathway, cytosolic DNA-sensing pathway, PI3K-Akt and other signaling pathways (Fig. 3A). Autophagy mediates recycling of cellular components by a series of autophagic vesicles formed with a membrane bound LC3B-II protein [3, 4, 34]. The autophagy associated genes were significantly activated after Drp1 overexpression in western blot analyses (Fig. 3B). In KYSE-30 cells, we found that elevated Drp1 expression significantly increased the LC3B-II protein levels and slightly reduced the levels of SQSTM1/p62 (Fig. 3C), suggesting mitochondria perturbation by abnormal Drp1 expression may modulate autophagy. Moreover, TEM analyses found elevated Drp1 expression increased the number of autophagic vesicles in KYSE-30 cells (Fig. 3D). In live cell imaging of GFP-LC3B expressing KYSE-30 cells, elevated Drp1 expression significantly increased the accumulation of GFP-LC3B puncta (Fig. 3E). In KYSE-30 xenograft tumors, LC3B staining was significantly increased in Drp1 overexpression compared with tumors in the respective control group (Fig. 3F). IHC staining showed that the level of the autophagy marker LC3B was significantly increased in ESCC patient tumor tissues than peritumor tissues (Fig. 3G), suggesting autophagy may play a role in ESCC. Furthermore, treatment with the autophagy inhibitor chloroquine significantly decreased their colony formation ability and the percentage of EdU positive cells in KYSE-30 with Drp1 overexpression (Fig. 3H and I), supporting a theme that autophagy is important for tumor cell proliferation and growth after Drp1 overexpression. In addition, Mdivi-1 significantly alleviated Drp1-mediated autophagy as shown by reversing the expression of LC3B-II and SQSTM1/p62, reducing accumulation of GFP-LC3B puncta and the formation of autophagic vesicles (Fig. 3C-E).

### cGAS-STING signaling pathway is involved in Drp1 overexpression-mediated autophagy

**Fig. 4:**
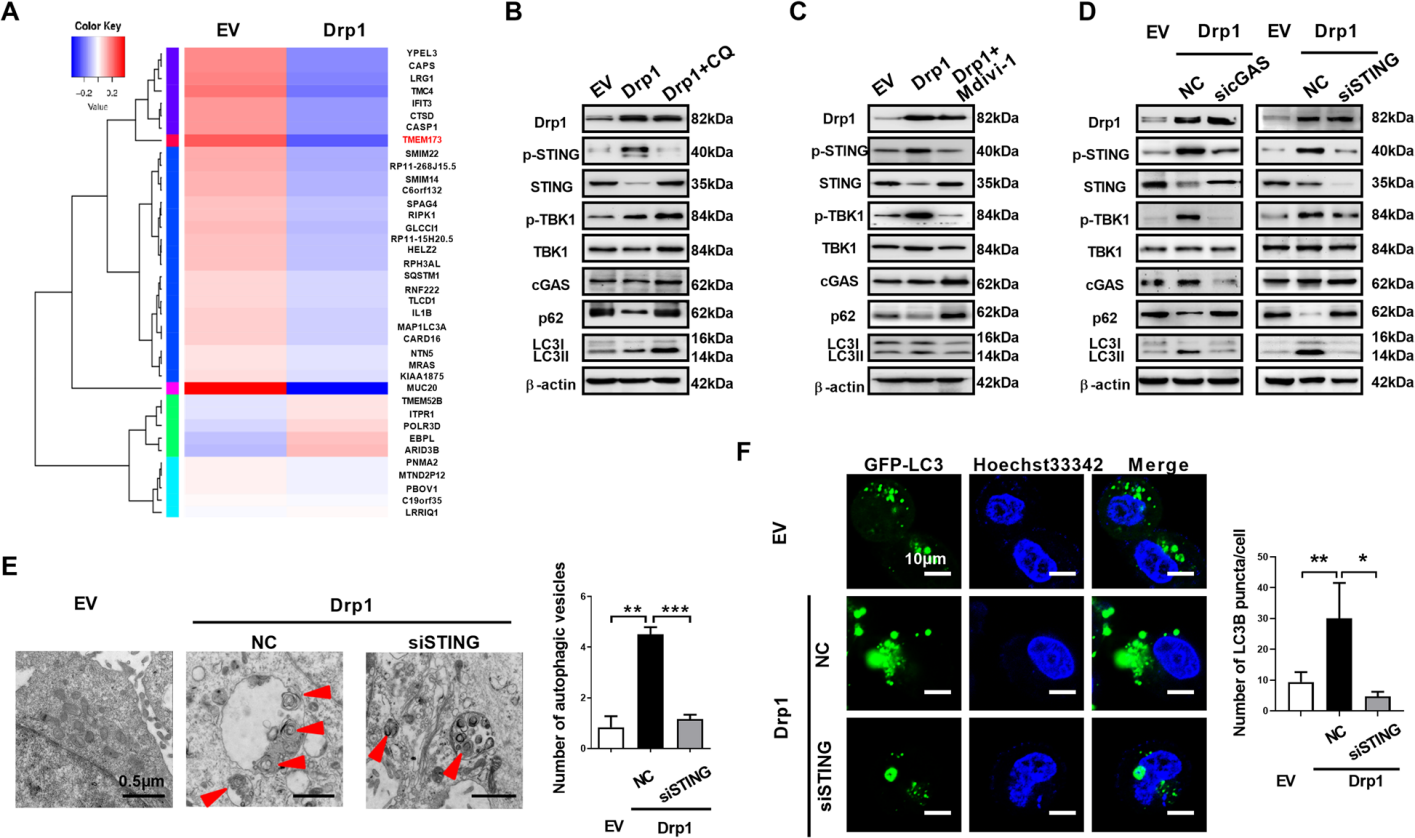
**cGAS-STING signaling pathway is involved in Drp1 overexpression-mediated autophagy. (A)** Heat map analysis of gene expression in ESCC cells as indicated. **(B, C and D)** Western blot analyses were performed in ESCC cells as indicated with antibodies specific for the proteins shown. **(E)** Representative transmission electron microscopy images of double-membrane autophagosomes in ESCC cells as indicated. Scale bar: 0.5 μm. **(F)** Representative images of fluorescent LC3B puncta (green) in ESCC cells as indicated. Scale bar: 10 μm. The data shown are the mean ± SEM from three separate experiments. Drp1, expression vector encoding Drp1; EV, empty vector; NC, negative control; CQ, chloroquine; siSTING, siRNAs against STING; sicGAS, siRNAs against cGAS.* P* value from *t* tests. **, *P* < 0.01; ***, *P* < 0.001

We and other laboratories previously showed that Drp1 overexpression markedly induces cytosolic mtDNA stress, which subsequently activates the cGAS-STING or TLR9 pathway [2, 12]. Recently, the cGAS-STING signaling pathway was found to promote autophagy [35, 36]. Hence, we investigated whether cGAS-STING signaling pathway is involved in Drp1 overexpression-mediated autophagy. Interestingly, we found TMEM173, which encoding STING, is downregulated expression after Drp1 overexpression (Fig. 4A). Western blot shown an increase in the phosphorylation of STING and TBK1 after Drp1 overexpression (Fig. 4B), indicating that the cGAS-STING pathway is activated. Further, the total amount of the STING protein is decreased, suggesting that STING might undergo autophagy mediated degradation (Fig. 4B). Upon chloroquine treatment to inhibit the formation of autolysosomes, the amount of STING protein was restored roughly to the same level with the control cells (Fig. 4B), supporting that STING was degradation by autophagy after the alternated expression of Drp1. Consistently, Mdivi-1 also inhibited STING pathway activation which induced by Drp1 overexpression (Fig. 4C). Moreover, knockdown of cGAS, an upstream activator of STING phosphorylation, decreased LC3B lipidation, attenuated STING and SQSTM1/p62 degradation, suppressed phosphorylation STING and TBK1 in cells with Drp1 overexpression (Fig. 4D). Furthermore, knockdown of STING blocked SQSTM1/p62 degradation, TBK-1 phosphorylation and LC3B conversion in cells with Drp1 overexpression (Fig. 4D). In addition, knockdown of STING with siRNAs in Drp1 overexpression significantly alleviated the autophagic vesicles formation (Fig. 4E) and accumulation of GFP-LC3B puncta (Fig. 4F). Together, these results indicate that cGAS-STING signaling pathway is involved in Drp1 overexpression-mediated autophagy in ESCC.

### Drp1 overexpression causes mitochondrial dysfunction and cytosolic mtDNA stress in ESCC cells

**Fig. 5:**
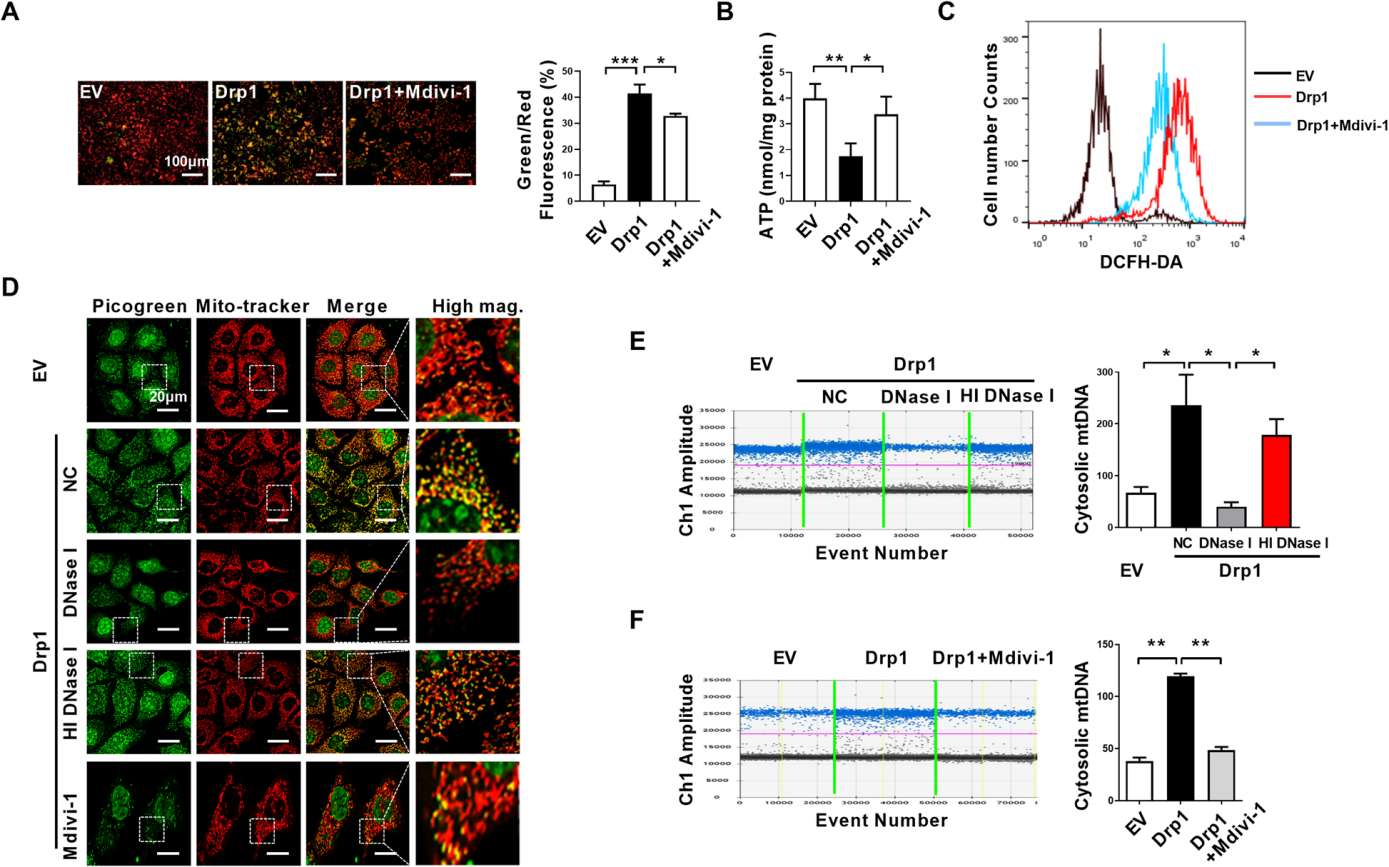
**Drp1 overexpression causes mitochondrial dysfunction and cytosolic mtDNA stress in ESCC cells. (A)** Mitochondrial membrane potential was analyzed by JC-1 staining in ESCC cells as indicated. Scale bars: 100 μm. **(B)** ATP concentration was measured in ESCC cells as indicated. **(C)** ROS production was measured with flow cytometry in ESCC cells as indicated. **(D)** Confocal microscopy images of indicated ESCC cells stained with Picogreen (green, DNA) and MitoTracker (red, mitochondria). Scale bar: 20 μm.**(E and F)** Cytosolic mtDNA content was measured using droplet digital PCR in ESCC cells as indicated. The data shown are the mean ± SEM from three separate experiments. Drp1, expression vector encoding Drp1; EV, empty vector; NC, negative control; HI DNase I, heat inactivated DNase I.* P* value from *t* tests. *, *P* < 0.05; **, *P* < 0.01; ***, *P* < 0.001

RNA sequencing indicated the activation of cytosolic DNA-sensing pathway (Fig. 3A). According our previously found, the cGAS-STING pathway activation by Drp1 overexpression prompted us to test if abnormal Drp1 expression regulates mtDNA release into the cytosol in ESCC. We fist investigated the mitochondrial function after Drp1 overexpression in ESCC cells. We observed that Drp1 overexpression decreased mitochondrial membrane potential detected with the JC-1 fluorescent molecule staining (Fig. 5A). In addition, Drp1 overexpression significantly reduced ATP production (Fig. 5B) and increased cellular reactive oxygen species (ROS) as detected with DCFH-DA staining in flow cytometry analyses (Fig. 5C). In contrast, Mdivi-1 treatment markedly retarded mitochondrial outer membrane permeabilization and ATP depletion, suppressed ROS production in cells with Drp1 overexpression (Fig. 5A-C). These results indicate abnormal expression Drp1 may disrupt mitochondrial homeostasis and result in mitochondrial dysfunction.

Moreover, Drp1 overexpression disorganized the mitochondrial nucleoid architecture and the amount of picogreen DNA staining areas without overlapping with the MitoTracker-red staining was increased (Fig. 5D). To analyze the cytosolic mtDNA concentration, the amount of mtDNA in the cytosol fraction lacking mitochondria was quantified using droplet digital PCR. Drp1 overexpression markedly increased mtDNA content as indicated by the increase in mitochondrial ND1 gene DNA in the cytosol than control cells (Fig. 5E). After DNase I protein was delivered into the cells, the amount of cytosolic mtDNA was significantly decreased using picogreen staining or droplet digital PCR analyses, while heat inactivated DNase I did not affect cytosolic mtDNA content (Fig. 5D and E). Mdivi-1 treatment also prevented Drp1 overexpression-induced cytosolic mtDNA release (Fig. 5D and F).

### Drp1 overexpression-induced cytosolic mtDNA stress is dependent on mPTP

**Fig. 6:**
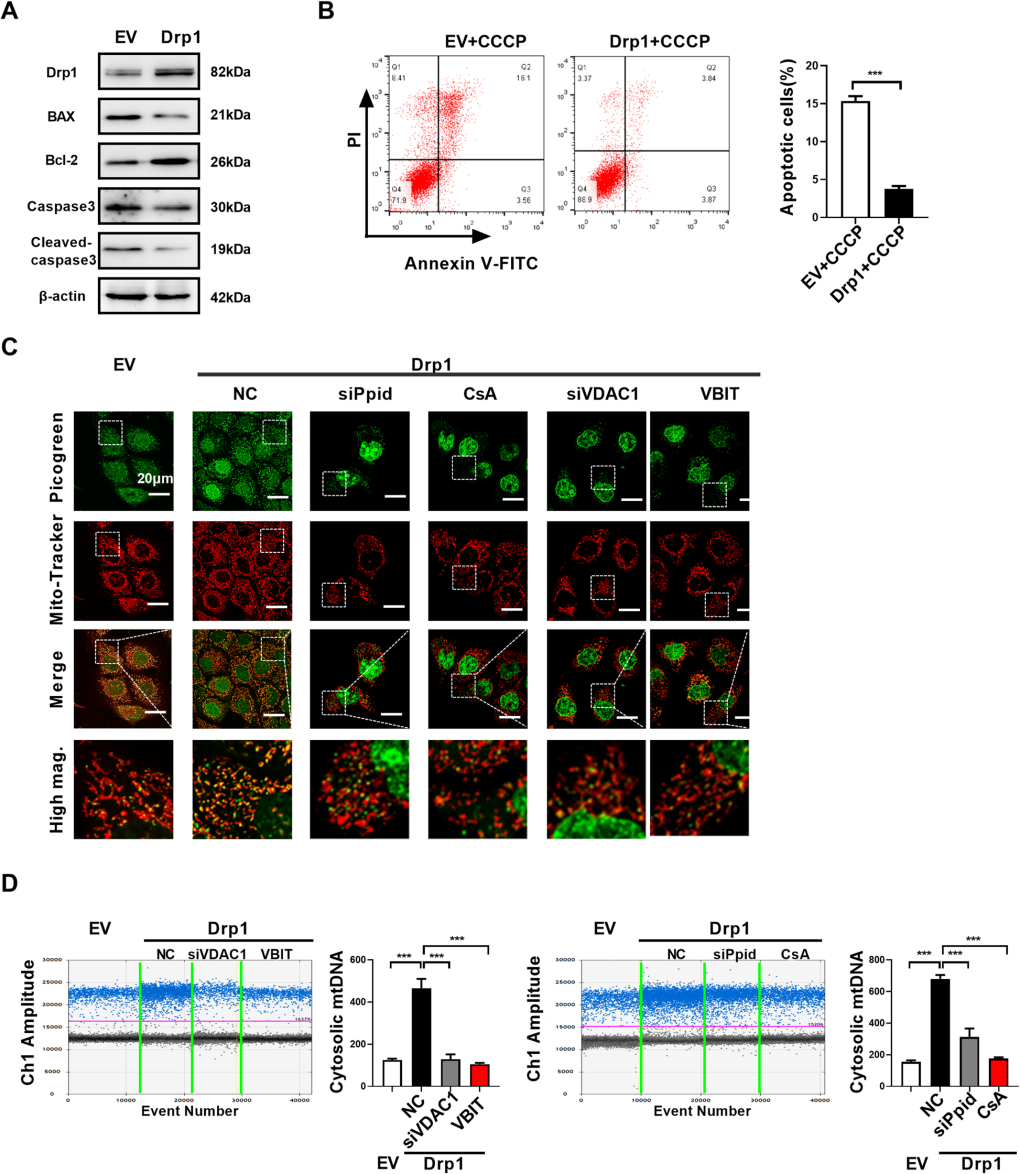
**Drp1 overexpression-induced cytosolic mtDNA stress is dependent on mPTP. (A)** Western blot analyses were performed in ESCC cells as indicated with antibodies specific for the proteins shown.**(B)** Flow cytometry analysis of apoptosis by Annexin V and PI staining in ESCC cells as indicated. The cells were treated with CCCP (150 mM) for 4 h before apoptosis analysis. **(C)** Confocal microscopy images of indicated ESCC cells stained with Picogreen (green, DNA) and MitoTracker (red, mitochondria). Scale bar: 20 μm. **(D)** Cytosolic mtDNA content was measured using droplet digital PCR in ESCC cells as indicated. The data shown are the mean ± SEM from three separate experiments. Drp1, expression vector encoding Drp1; EV, empty vector. siPpid, siRNAs against Ppid; siVDAC1, siRNAs against VDAC1; CsA, cyclosporin A; VBIT, VDAC1 oligomerization inhibitor VBIT-4.* P* value from *t* tests. ***, *P* < 0.001

It has been reported that mitochondrial permeability transition pore (mPTP), BAX/BAK macropores, voltage-dependent anion channel 1 (VDAC1) oligomerization, and/or the formation of globular mitochondria are involved in mediating mtDNA release under different cell stress [26, 27, 37, 38]. BAX/BAK macropores is appeared in mitochondrial outer membrane after BAK and BAX oligomerization during apoptosis and allows mtDNA to be exposed to cytoplasm [38]. However, in this study, we found that Drp1 overexpression inhibited the apoptosis of ESCC cells, as indicated by downregulation of BAX/Bcl-2 ratio and cleaved caspase-3 (Fig. 6A). In addition, we observed that Drp1 overexpression significantly inhibited CCCP-induced apoptosis of ESCC cells (Fig. 6B). Instead, our results observed loss of membrane potential (Fig. 5A) and upregulation of ROS (Fig. 5C), which indicated opening of the mitochondrial permeability transition pore (mPTP) [39]. Previous research had reported that Mdivi-1 directly inhibited mitochondrial outer membrane permeabilization [40], which was demonstrated by our results (Fig. 5A). Additionally, downregulated expression of Ppid using siRNA (encoding the mPTP component PPID) or pharmacological inactivation of the mPTP using cyclosporin A (CsA) prevented Drp1 overexpression-mediated mtDNA release into the cytoplasm (Fig. 6C and D). VDAC1 is another component that may participate in cytosolic release of mtDNA under oxidative stress [37]. Agreement with our speculated, Drp1 overexpression-mediated cytosolic mtDNA accumulation is also significantly inhibited after treatment with VDAC1 oligomerization inhibitor VBIT-4 or siRNA of VDAC1 (Fig. 6C and D). Together, these data indicated that Drp1 overexpression-induced cytosolic mtDNA stress is dependent on opening the mPTP and VDAC1 oligomerization.

### Cytosolic mtDNA stress mediates the cGAS-STING signaling activation, autophagy induction, and cell proliferation of ESCC

**Fig. 7:**
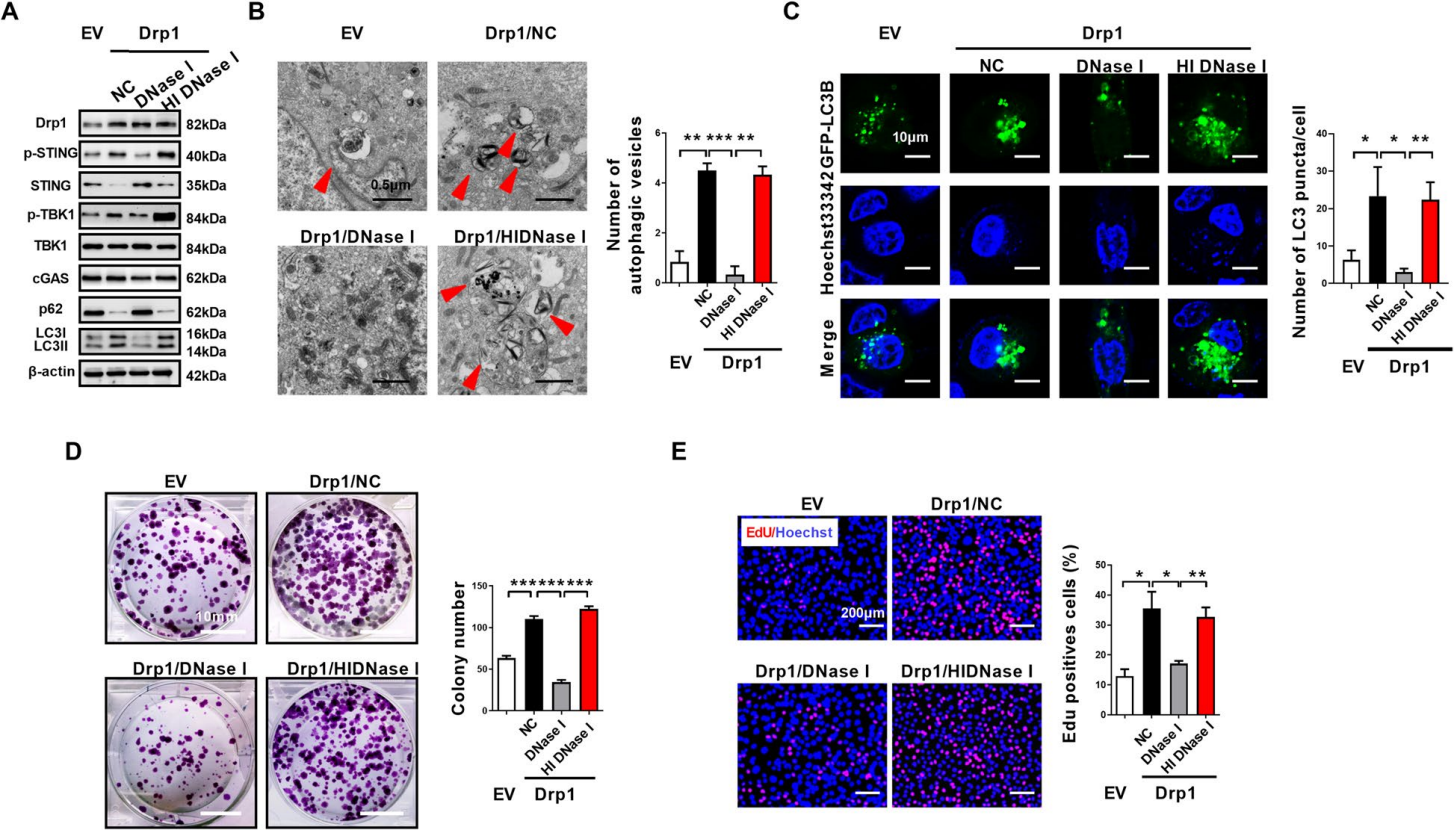
**Cytosolic mtDNA stress mediates the cGAS-STING signaling activation, autophagy induction, and cell proliferation of ESCC. (A)** Western blot analyses were performed in ESCC cells as indicated with antibodies specific for the proteins shown.**(B)** Representative transmission electron microscopy images of double-membrane autophagosomes in ESCC cells as indicated. Scale bar: 0.5 μm. **(C)** Representative images of fluorescent LC3B puncta (green) in ESCC cells as indicated. Scale bar: 10 μm. **(D)** Colony-forming potential were detected in KYSE-30 cells as indicated. Scale bar: 10 mm. **(E)** Cell proliferation was evaluated by EdU incorporation assay in KYSE-30 cells as indicated. Scale bar: 200 μm. The data shown are the mean ± SEM from three separate experiments. Drp1, expression vector encoding Drp1; EV, empty vector; NC, negative control; HI DNase I, heat inactivated DNase I.* P* value from *t* tests. *, *P* < 0.05; **, *P* < 0.01; ***, *P* < 0.001

Consistent with a role of cytosolic mtDNA in the activation of the cGAS-STING pathway, we found DNase I treatment but not the heat inactivated DNase I treatment attenuated STING phosphorylation after Drp1 overexpression in ESCC cells (Fig. 7A). Moreover, DNase I treatment also blocked LC3B lipidation and the formation of autophagic vesicles (Fig. 7A and B), suggesting cytosolic mtDNA plays an important role in activating the cGAS-STING signaling pathway and autophagy induction. Furthermore, we found treatment with active DNase I but not heat inactivated DNase I significantly decreased Drp1 overexpression-mediated accumulation of GFP-LC3B puncta (Fig. 7C). Additionally, the colony formation ability and EdU incorporation of ESCC cells with Drp1 overexpression are markedly ameliorated after DNase I treatment (Fig. 7D and E). These results suggested that cytosolic mtDNA plays a direct role in cGAS-STING signaling activation, autophagy induction and proliferation of ESCC cells.

### Blocking cGAS-STING pathway inhibits mtDNA stress induced ESCC progression.

**Fig. 8:**
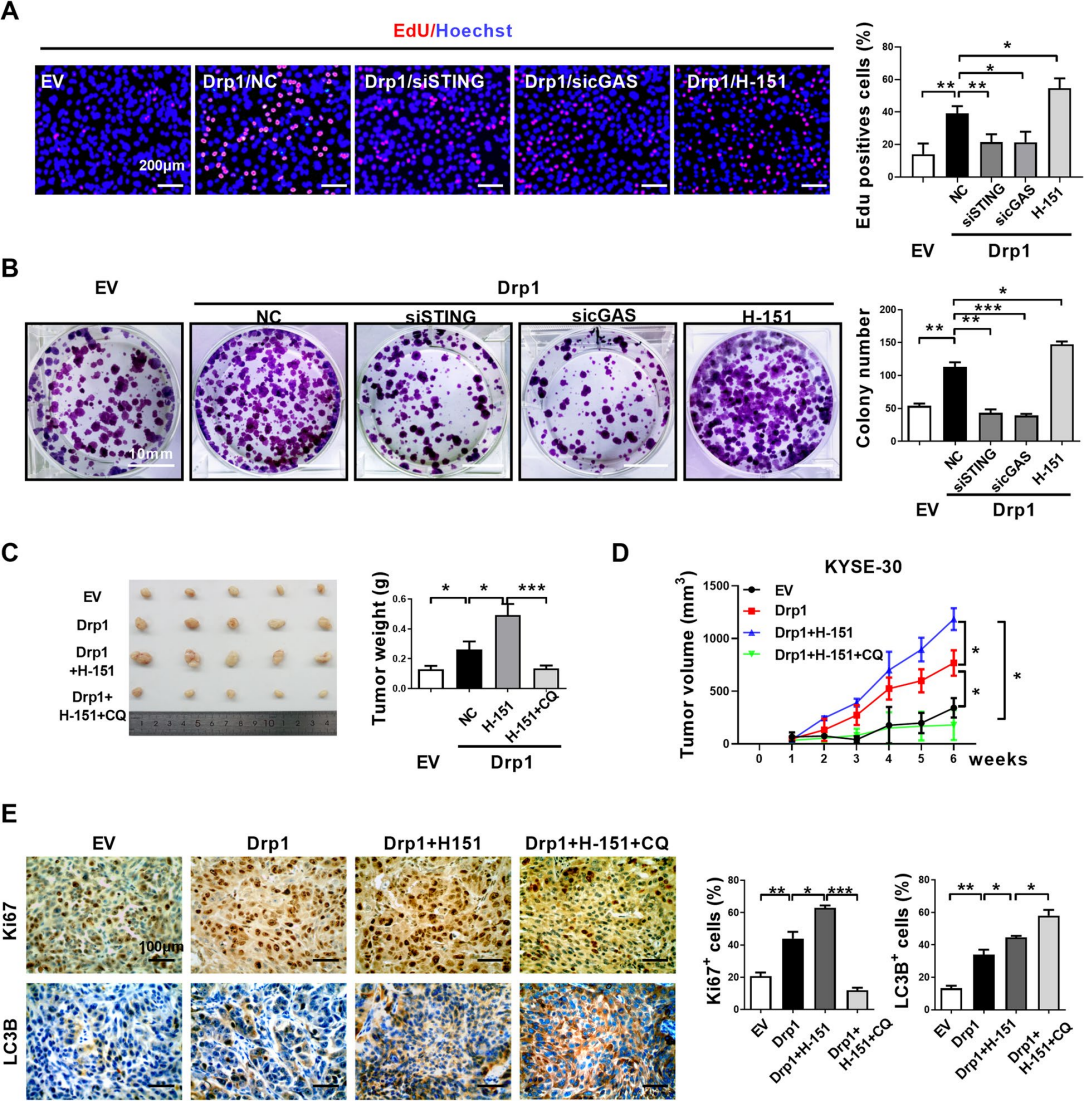
**Blocking cGAS-STING pathway inhibits mtDNA stress induced ESCC progression. (A)** Cell proliferation was evaluated by EdU incorporation assay in KYSE-30 cells treatment with indication. Scale bar: 200 μm. **(B)** Colony-forming potential were detected in KYSE-30 cells treatment with indication. Scale bar: 10 mm. **(C and D)** Tumor growth curves and weight of subcutaneous xenograft tumor model developed from ESCC cells treatment with indication (*n* = 5). **(E)** Representative IHC staining images of Ki67 and LC3B in xenograft tumors developed from KYSE-30 cells treatment with indication. Scale bar: 100 μm. The data shown are the mean ± SEM from three separate experiments. Drp1, expression vector encoding Drp1; EV, empty vector; NC, negative control; siSTING, siRNAs against STING; sicGAS, siRNAs against cGAS; CQ, chloroquine. *P* value from *t* tests. *, *P* < 0.05; **, *P* < 0.01; ***, *P* < 0.001

To test the effect of autophagy mediated by cGAS-STING pathway on Drp1 overexpression-induced ESCC progression, we analyzed cells survival in ESCC cells treated with cGAS or STING siRNAs. We observed that Drp1 overexpression-induced EdU incorporation and proliferative potential were significantly attenuated in cGAS or STING knockdown cells as compared to control groups (Fig. 8A and B). Further, we evaluated the cellular effect of STING inhibitor H-151 in Drp1 overexpression-induced ESCC cells survival. Unexpectedly, H-151 slightly increased EdU incorporation and the colony formation ability in Drp1 overexpression cells (Fig. 8A and B). In xenograft experiments, H-151 treatment slightly increased tumor weight, tumor growth rate and the percentages of Ki67^+^ cells in tumor samples (Fig. 8C and D). In contrast, chloroquine treatment significantly decreased the promotional effect of H-151 on xenograft tumor growth and cell proliferation in Drp1 overexpression or TFAM depletion (Fig. 8C and D).

In IHC analyses of xenograft tumors, elevated LC3B staining was observed after treatment with H-151 than tumors with Drp1 overexpression, while treatment with the autolysosome formation inhibitor chloroquine further increased the LC3B accumulation (Fig. 8E). In Drp1 overexpression cells, H-151 treatment significantly reduced TBK1 phosphorylation and suppressed IFN-β expression (Fig. S6A and B), which is consistent with a role of STING in the phosphorylation of TBK-1 and the type I interferon gene expression. However, inhibition of STING by H-151 did not block STING pathway-mediated autophagy, as indicated by further STING and SQSTM1/p62 degradation (Fig. S6A), as well as the accumulation of autophagic vesicles in TEM assays and GFP-LC3B speckle formation analyses (Fig. S6C and D). These results suggest that upon activation by mtDNA, STING plays two distinct roles in promoting autophagy and type I interferon gene expression, and that STING inhibition with H-151 is not sufficient to block the autophagy function of STING hence may further increase tumor growth.

## Discussion

**Fig. 9:**
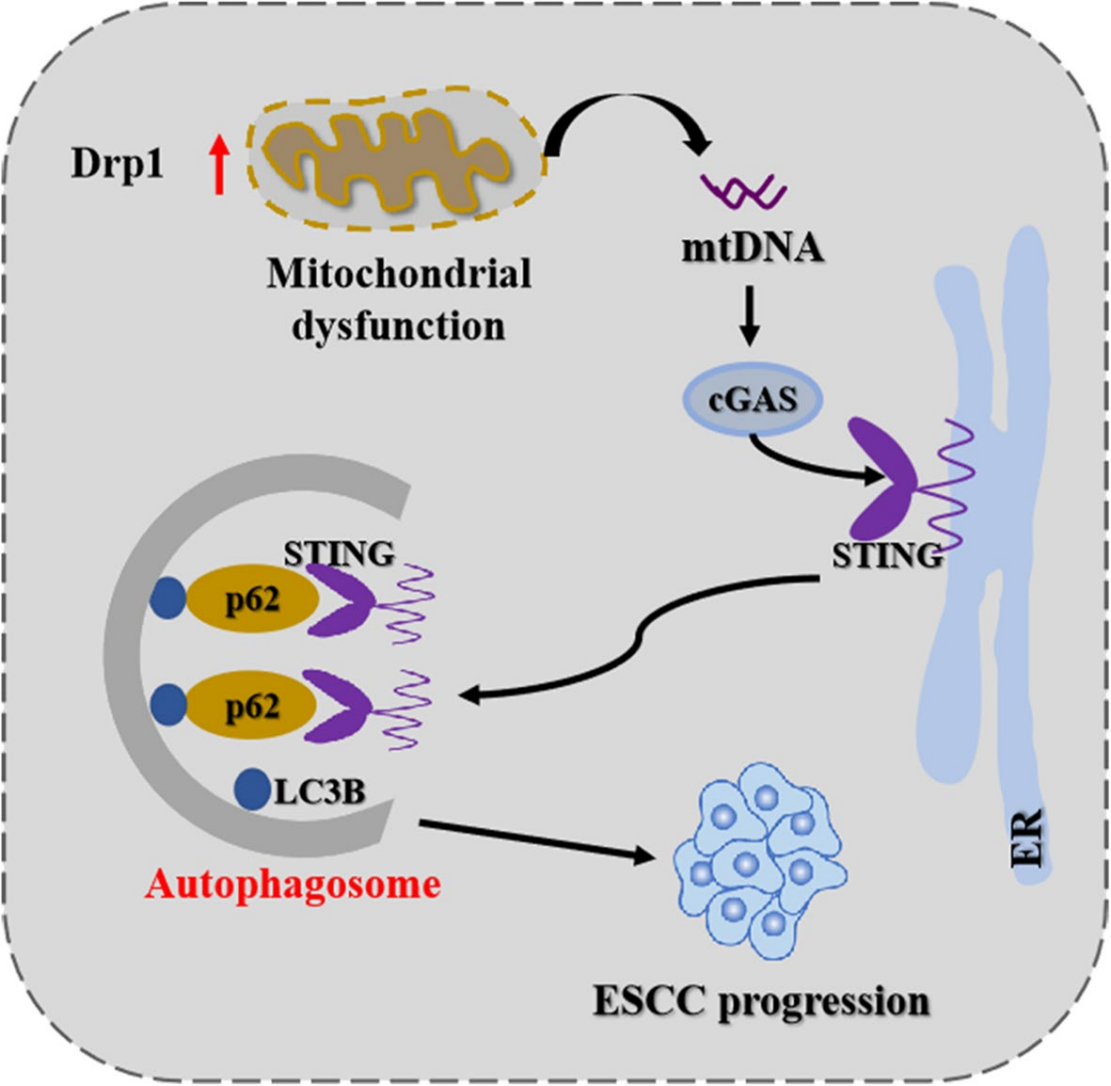
Schematic depicting the effect of mitochondrial fission-induced mtDNA stress promotes ESCC progression through cGAS-STING pathway mediated autophagy

Accumulating evidence indicates that mitochondrial dynamics homeostasis is involved in tumor progression [3, 4]. However, the role of Drp1 in ESCC progression has not been systematically elucidated. Here, we demonstrated for the first time that a high expression of Drp1 is correlated with poor overall survival of ESCC patients. Drp1 overexpression significantly induces mitochondrial dysfunction and cytosolic mtDNA stress, which subsequently activates the cGAS-STING pathway and triggers autophagy, consequently promotes ESCC cancer growth (Fig. 9).

Previous studies had shown that mitochondria played a central and multi-functional role in tumor progression by modulating mitochondrial mass, dynamics and biogenesis [3, 4]. Consistent with previous findings, we found that upregulated Drp1 (the critical GTPase for dynamic) significantly induced mitochondrial dysfunction in ESCC cells, which is commonly indicated by decreased ATP generation and mitochondrial membrane potential, and increased ROS production. Previous studies had shown that Drp1 was implicated in tumor progression [3, 4, 34]. Here, our data demonstrated that Drp1 overexpression promotes survival of ESCC in vitro and in tumor xenograft models, suggesting a critical role of mitochondrial dynamic homeostasis in ESCC tumor progression.

Mitochondrial dynamics and biogenesis play important roles in nucleoid homeostasis and cristae reformation [1, 8]. Our previous study demonstrated that increased mitochondrial fission by Drp1 overexpression may disrupt nucleoid structure and induce cytosolic mtDNA stress in HCC cells [12]. Consistent with previous reports, we found that the architecture of mitochondrial nucleoids was significantly disorganized and cytosolic mtDNA content was markedly increased in Drp1 overexpression ESCC cells. In addition, depletion of cytosolic mtDNA after DNase I treatment significantly decreased ESCC cell growth, EdU incorporation, and xenograft tumor growth, suggesting that mtDNA release to the cytosol is important for Drp1-mediated cancer cell growth. The molecular mechanism underlying mitochondrial DNA release into the cytosol is largely unknown. Mitochondrial permeability transition pore (mPTP), BAX/BAK macropores, VDAC1 oligomerization, and/or the formation of globular mitochondria are involved in mediating mtDNA release under different cell stress [26, 27, 37, 38]. Our results found that Drp1 may regulate mtDNA release through opening the mPTP and VDAC1 oligomerization in ESCC cells. Certainly, further studies should be performed to reveal the underling molecular mechanisms by which mitochondrial dysfunction mediates the cytosolic mtDNA tress.

Previous study demonstrated that cytosolic mtDNA is involved in STING signaling-mediated autophagy and antiviral innate immune response [35, 36]. Our results indicated that Drp1 overexpression-induced cytosolic mtDNA stress dramatically upregulates the phosphorylation of TBK1 and STING, and stimulates the formation of autophagic vesicles and protein turnover. Conversely, mtDNA depletion significantly abrogated autophagosomes formation and GFP-LC3B fluorescence puncta accumulation in ESCC cells with Drp1 overexpression. We further found that autophagy inhibitors markedly alleviated Drp1 overexpression-induced survival of ESCC cells. It has been demonstrated that activation of the cGAS-STING pathway plays a dichotomous role in tumor development [22, 24]. Some research has demonstrated that activation of STING signaling also promoted autophagy [35, 36]. However, whether the autophagy mediated by STING pathway contributes to tumor progression remain unexplored. In this study, we found that STING mediated autophagy plays a role in Drp1 overexpression-induced ESCC cells survival. Blocking cGAS-STING pathway significantly abrogated autophagy and ESCC survival induced by Drp1 overexpression. Surprisingly, H-151, a potent and selective covalent antagonist of STING, failed to suppress Drp1 overexpression-induced ESCC survival. Conversely, H-151 slightly increased the ESCC cell growth. We found that H-151 only inhibited STING-mediated immune responses by inhibiting TBK1 phosphorylation and IFN-β expression but not autophagy. Furthermore, we found that combination chloroquine with H-151 remarkably repressed subcutaneous tumor growth capacity of ESCC cells with Drp1 overexpression. Collectively, these findings indicate STING-mediated autophagy pathway promotes ESCC cells survival under mtDNA stress conditions. Our results raise concerns about the usage of the STING inhibitors as drugs for ESCC patients. Instead, targeted suppression of STING-mediated autophagy and simultaneous induction of STING-mediated immune response serve as better therapeutic strategy for cancer patients.

## Conclusions

In present study, we observed a cytosolic mtDNA stress after Drp1 overexpression-induced mitochondrial dysfunction, and subsequent activation of the cGAS-STING pathway and induction of autophagy in ESCC cells (Fig. 9). Our results suggest an important contribution of the STING pathway to cytosolic mtDNA stress-induced autophagy, and targeted suppression of STING-mediated autophagy as a therapeutic strategy for ESCC.

## Supplementary Information

**Additional file 1.**

## Data Availability

All data are available in the main text or the supplementary materials. Sequencing data have been deposited in GEO under accession No. GSE182710.

## Abbreviations

mtDNA: Mitochondrial DNA
ddPCR: Droplet digital PCR
siRNA: Small interfering RNA
qRT-PCR: Quantitative real-time polymerase chain reaction
shRNA: Short hairpin RNA
CQ: Chloroquine
CsA: Cyclosporin A
ROS: Reactive oxygen species
IFNs: Type I interferons
cGAS: Cyclic GMP-AMP synthase
STING: Stimulator of interferon genes
Drp1: The critical GTPase for mitochondrial dynamics
ESCC: Esophageal squamous cell carcinoma
RNA-seq: RNA sequencing
