# Supplementary material for: Increased Drp1 promotes autophagy and ESCC progression by mtDNA stress mediated cGAS-STING pathway
Source: J Exp Clin Cancer Res. 2022 Feb 24;41:76. doi: 10.1186/s13046-022-02262-z (PMC8867650; doi:10.1186/s13046-022-02262-z)
Supplement: Supplementary file 1 — Additional file 1. [file 13046_2022_2262_MOESM1_ESM.docx]

**Supplementary Material**

**Increased Drp1 promotes autophagy and ESCC progression by mtDNA stress mediated cGAS-STING pathway**

**Li *et al.***

**Supplementary Figure legends**

**Supplementary Figure S1. Drp1 regulate mitochondrial morphology in ESCC cells.**

Western blot **(A, C, E)** and qRT-PCR **(B, D, F)** analyses for Drp1 expression were performed in ESCC cells, which were stably transfected with expression vector as indicated. **(G and H)** Confocal microscopy analysis of mitochondrial network in different ESCC cells as indicated. Scale bars: 10 μm. The data shown are the mean ± SEM from three independent experiments. Drp1, expression vector encoding Drp1; EV, empty vector. *P* value from *t* tests. *, *P* < 0.05; **, *P* < 0.01; ***, *P* < 0.001.

**Supplementary Figure S2. Drp1-mediated mitochondrial fission promoted growth of ESCC cells *in vitro*.**

**(A)** Cell viability of EC9706 ESCC cells with Drp1 overexpression were evaluated using the MTS cell proliferation assay. **(B)** Cell proliferation was evaluated by EdU incorporation assay in EC9706 ESCC cells as indicated. Scale bar: 200 μm. **(C)** Colony-forming potential were detected in EC9706 ESCC cells with treatment as indicated. Scale bar: 10 mm. The data shown are the mean ± SEM from three separate experiments. *, *P* < 0.05; **, *P* < 0.01; ***, *P* < 0.001.

**Supplementary Figure S3. Suppression of Drp1 promotes mitochondrial elongated in KYSE-70 ESCC cells.**

Western blot **(A)** and qRT-PCR **(B)** analyses for Drp1 expression were performed in KYSE-70 ESCC cells as indicated. (**C and D)** Confocal microscopy analysis of the mitochondrial network in ESCC cells treated with shDrp1 or 50μM Mdivi-1 for 12 h as indicated. Scale bar: 10 μm. The data shown are the mean ± SEM from three independent experiments. shDrp1, shRNA expression vector against Drp1; shCtrl, control shRNA. *P* value from *t* tests. *, *P* < 0.05; **, *P* < 0.01.

**Supplementary Figure S4. Suppression of Drp1 promotes mitochondrial elongated in KYSE-140 ESCC cells.**

Western blot **(A)** and qRT-PCR **(B)** analyses for Drp1 expression were performed in KYSE-140 ESCC cells as indicated. (**C and D)** Confocal microscopy analysis of the mitochondrial network in ESCC cells treated with shDrp1 or 50μM Mdivi-1 for 12 h as indicated. Scale bar: 10 μm. The data shown are the mean ± SEM from three independent experiments. shDrp1, shRNA expression vector against Drp1; shCtrl, control shRNA. *P* value from *t* tests. ***, *P* < 0.001.

**Supplementary Figure S5. Suppression of Drp1-mediated mitochondrial fission inhibited growth of KYSE-140 ESCC cells *in vitro*.**

**(A and B)** Cell viability of KYSE-140 ESCC cells with Drp1 knockdown or treatment with 50 μM Mdivi-1 for 12 h as indicated were evaluated using the MTS cell proliferation assay. **(C and D)** Colony-forming potential were detected in KYSE-140 ESCC cells with Drp1 knockdown or treatment with 50 μM Mdivi-1 for 12 h as indicated. Scale bar: 10 mm. **(E and F)** Cell proliferation was evaluated by EdU incorporation assay in KYSE-140 ESCC cells with Drp1 knockdown or treatment with 50 μM Mdivi-1 for 12 h as indicated. Scale bar: 200 μm. *, *P* < 0.05; **, *P* < 0.01; ***, *P* < 0.001.

**Supplementary Figure S6. The effect of H151 on cGAS-STING signaling pathway-mediated autophagy.**

**(A)** Western blot analyses were performed in ESCC cells as indicated with antibodies specific for the proteins shown. **(B)** qRT-PCR analyses for expression levels of IFN-β in ESCC cells as indicated. **(C)** Representative transmission electron microscopy images of double-membrane autophagic vesicles in ESCC cells as indicated. Scale bar: 0.5 μm. **(D)** Representative images of fluorescent LC3B puncta (green) in ESCC cells as indicated. Scale bar: 10 μm. The data shown are the mean ± SEM from three independent experiments. Drp1, expression vector encoding Drp1; EV, empty vector; NC, negative control. *P* value from *t* tests. *, *P* < 0.05.

**Supplementary Tables**

**Supplementary Table S1. Distribution of ESCC patients’ characteristics.**

| **Variable** | **All patients, n (%), Total = 61** |
| --- | --- |
| Sex |  |
| Female | 15 (24.6%) |
| Male | 46 (75.4%) |
| Age, years |  |
| <60 | 27 (44.3%) |
| ≥60 | 34 (55.7%) |
| T classification |  |
| T1 | 17 (27.9%) |
| T2 | 26 (42.6%) |
| T3 | 18 (29.5%) |
| N classification |  |
| Absent (0) | 40 (65.57%) |
| Present (1/2/3) | 21 (34.43%) |
| M classification |  |
| M0 | 53 (86.89%) |
| M1 | 8 (13.11%) |
| Histological grade |  |
| G1 | 15 (24.59%) |
| G2 | 38 (62.30%) |
| G3 | 8 (13.11%) |
| Size of tumor, cm |  |
| <5cm | 52 (85.25%) |
| ≥5cm | 9 (14.75%) |
| Tumor location |  |
| Lower thoracic esophagus | 9 (14.75%) |
| Middle thoracic esophagus | 47 (77.05%) |
| Upper thoracic esophagus | 5 (8.20%) |
| Survival |  |
| Death | 20 (32.79%) |
| Alive | 41 (67.21%) |

**Supplementary Table S2. Sequence of primers and recombinant DNA、siRNAs.**

| 1. **Primers used in qPCR analyses** | | |
| --- | --- | --- |
| Drp1 | Forward primer | AAGGAGCCAGTCAAATTATTGC |
|  | Reverse primer | AGTCAACAAAGTCTCAGTATTA |
| ND1 | Forward primer | CCCTAAAACCCGCCACATCT |
|  | Reverse primer | GAGCGATGGTGAGAGCTAAGGT |
| GAPDH | Forward primer | AACGGATTTGGTCGTATTGG |
|  | Reverse primer | TTGATTTTGGAGGGATCTCG |
| IFN-β | Forward primer | CATTACCTGAAGGCCAAGGA |
|  | Reverse primer | CAATTGTCCAGTCCCAGAGG |
| ND1 Probe | CCATCACCCTCTACATCACCGCCC | |
| **2. Recombinant DNA**   \| **Recombinant DNA** \| **Source** \| **Identifier** \| \| --- \| --- \| --- \| \| pcDNA3.1(+)-GFP-LC3B \| GenePharma \| Cat# B5500 \| \| pcDNA3.1(+) \| This paper \| N/A \| \| pcDNA3.1(+)-Drp1 \| This paper \| N/A \| \| pSilencer™ 3.1-H1 neo \| This paper \| N/A \| \| pSilencer™3.1-H1 neo-shDrp1 \| This paper \| N/A \|   **3. siRNAs** | | |
| Drp1 siRNA | Sense primer | ACUAUUGAAGGAACUGCAAAAUAUATT |
|  | Antisense | UAUAUUUUGCAGUUCCUUCAAUAGUTT |
| cGAS siRNA | Sense primer | CCAACACUCGUGCAUAUUATT |
|  | Antisense | UAAUAUGCACGAGUGUUGGTT |
| STING siRNA | Sense primer | GCCCGGAUUCGAACUUACAAUTT |
|  | Antisense | AUUGUAAGUUCGAAUCCGGGCTT |
| TBK1 siRNA | Sense primer | GGGAACCUCUGAAUACCAUTT |
|  | Antisense | AUGGUAUUCAGAGGUUCCCTT |
| Control siRNA | Sense primer | UUCUCCGAACGUGUCACGUTT |
|  | Antisense | ACGUGACACGUUCGGAGAATT |

**Supplementary Table S3. Antibodies used in this manuscript.**

| **Antibodies for Western Blot** | **Source** | **Identifier** |
| --- | --- | --- |
| Mouse Anti-DNM1L monoclonal antibody | Abcam | Cat# ab56788;RRID:AB_941306 |
| Rabbit Anti-LC3B (D11) XP monoclonal antibody | Cell Signaling | Cat# 3868; RRID:AB_2137707 |
| Rabbit Anti-SQSTM1/p62 (D5E2) monoclonal antibody | Cell Signaling | Cat# 8025; RRID:AB_10859911 |
| Rabbit Anti-STING (D2P2F) monoclonal antibody | Cell Signaling | Cat# 13647; RRID:AB_2732796 |
| Rabbit Anti-phospho-STING (Ser366) (D7C3S) monoclonal antibody | Cell Signaling | Cat# 19781;RRID:AB_2737062 |
| Rabbit Anti-NAK/TBK1 monoclonal antibody | Abcam | Cat# ab40676; RRID:AB_776632 |
| Rabbit Anti-phospho-NAK/TBK1 (Ser172) monoclonal antibody | Abcam | Cat# ab109272;RRID:AB_10862438 |
| Rabbit Anti-cGAS (D1D3G) monoclonal antibody | Cell Signaling | Cat# 15102;RRID:AB_2732795 |
| Mouse Anti-beta Actin monoclonal Antibody | Proteintech | Cat# 60008-1-Ig;RRID:AB_2289225 |
| BAX polyclonal Antibody | Proteintech | Cat# 5099-2-lg |
| BLC2 polyclonal Antibody | Proteintech | Cat# 12789-1-AP |
| Cleaved Caspase-3 | Cell Signaling | Cat# 9661S |
| Caspase-3 | Cell Signaling | Cat# 9662 |
| **Antibodies for Immunohistochemistry (IHC)** | | |
| Mouse Anti-DNM1L monoclonal antibody | Abcam | Cat# ab56788;RRID:AB_941306 |
| Rabbit Anti-LC3B (D11) XP monoclonal antibody | Cell Signaling | Cat# 3868; RRID:AB_2137707 |
| Rabbit Anti-Ki-67 (D3B5) monoclonal antibody | Cell Signaling | Cat# 12202; RRID: AB_2620142 |
| **Secondary Antibodies** | | |
| Anti-mouse IgG, HRP-linked Antibody | Cell Signaling | Cat# 7076; RRID: AB_330924 |
| Anti-rabbit IgG, HRP-linked Antibody | Cell Signaling | Cat# 7074; RRID: AB_2099233 |

| **Supplementary Table S4. Differentially expressed mRNAs in RNA sequencing data of Drp1 overexpression**  **(log2fold change>1, p<0.05)** | | | | | |
| --- | --- | --- | --- | --- | --- |
| **Gene ID** | **baseMean** | **log2Foldchange** | **pvalue** | **padj** | **Gene Symbol** |
| ENSG00000018236 | 558.598058 | 4.901219295 | 5.2E-252 | 8.66E-249 | CNTN1 |
| ENSG00000006756 | 120.2223552 | 4.872929817 | 1.52E-78 | 2.48E-76 | ARSD |
| ENSG00000185736 | 82.77108118 | 4.481928585 | 2.72E-62 | 3.1E-60 | ADARB2 |
| ENSG00000120675 | 50.19337911 | 3.849745111 | 9.55E-43 | 5.23E-41 | DNAJC15 |
| ENSG00000170571 | 108.8535839 | 3.741795243 | 4.43E-64 | 5.37E-62 | EMB |
| ENSG00000152661 | 453.525176 | 3.43576822 | 9.33E-189 | 8.55E-186 | GJA1 |
| ENSG00000161249 | 116.8885436 | 3.261695334 | 2.56E-59 | 2.56E-57 | DMKN |
| ENSG00000102109 | 91.35524413 | 3.067898265 | 2.44E-47 | 1.69E-45 | PCSK1N |
| ENSG00000115461 | 117.6960106 | 3.036734592 | 6.52E-55 | 5.63E-53 | IGFBP5 |
| ENSG00000119508 | 910.5905768 | 3.003425041 | 3.18E-284 | 7.28E-281 | NR4A3 |
| ENSG00000162817 | 41.28583644 | 2.871677947 | 3.52E-27 | 8.88E-26 | C1orf115 |
| ENSG00000116741 | 651.9308422 | 2.860139685 | 1.78E-206 | 1.82E-203 | RGS2 |
| ENSG00000243225 | 91.36224639 | 2.835719313 | 5.85E-42 | 3.12E-40 | RP11-7F17.1 |
| ENSG00000099284 | 43.45787828 | 2.751944521 | 3.82E-25 | 8.6E-24 | H2AFY2 |
| ENSG00000116711 | 63.92804849 | 2.664612911 | 5.59E-30 | 1.64E-28 | PLA2G4A |
| ENSG00000048740 | 174.5944046 | 2.580429893 | 1.56E-63 | 1.84E-61 | CELF2 |
| ENSG00000176907 | 122.4905699 | 2.548834401 | 2.71E-47 | 1.85E-45 | C8orf4 |
| ENSG00000118785 | 35.56199009 | 2.417110631 | 1.6E-19 | 2.54E-18 | SPP1 |
| ENSG00000164687 | 48.23669556 | 2.406644389 | 2E-22 | 3.76E-21 | FABP5 |
| ENSG00000212724 | 78.38122026 | 2.367085718 | 5.11E-30 | 1.5E-28 | KRTAP2-3 |
| ENSG00000116745 | 21.55132409 | 2.320589256 | 4.35E-15 | 5.07E-14 | RPE65 |
| ENSG00000103490 | 130.2619922 | 2.261414784 | 4.01E-42 | 2.15E-40 | PYCARD |
| ENSG00000112218 | 44.70809914 | 2.258068327 | 1.49E-19 | 2.38E-18 | GPR63 |
| ENSG00000078401 | 75.76736664 | 2.231110894 | 9.24E-26 | 2.17E-24 | EDN1 |
| ENSG00000166033 | 20.04856258 | 2.197075011 | 9.84E-14 | 1.03E-12 | HTRA1 |
| ENSG00000144583 | 33.39168577 | 2.167163786 | 3.09E-16 | 3.93E-15 | MARCHF4 |
| ENSG00000258602 | 219.0252708 | 2.150471757 | 3.11E-49 | 2.23E-47 | RP11-7F17.7 |
| ENSG00000151883 | 26.69606421 | 2.135047072 | 3.05E-14 | 3.32E-13 | PARP8 |
| ENSG00000065989 | 34.71909179 | 2.063741995 | 3.04E-15 | 3.59E-14 | PDE4A |
| ENSG00000151835 | 131.0328846 | 2.033801977 | 3.75E-37 | 1.61E-35 | SACS |
| ENSG00000198721 | 46.55882886 | 2.028498363 | 1.33E-16 | 1.72E-15 | ECI2 |
| ENSG00000128602 | 14.54088073 | 2.019691196 | 5E-11 | 4.05E-10 | SMO |
| ENSG00000169429 | 832.6862092 | 1.982972094 | 1.09E-37 | 4.78E-36 | IL8 |
| ENSG00000130600 | 10144.63275 | 1.976570604 | 0 | 0 | H19 |
| ENSG00000116141 | 185.8037779 | 1.973247041 | 1.48E-46 | 9.82E-45 | MARK1 |
| ENSG00000185565 | 33.35163256 | 1.97171324 | 5.15E-14 | 5.53E-13 | LSAMP |
| ENSG00000228966 | 15.87116746 | 1.949945561 | 9.95E-11 | 7.84E-10 | HOMER2P1 |
| ENSG00000134259 | 37.01395009 | 1.885906589 | 9.8E-14 | 1.02E-12 | NGF |
| ENSG00000183688 | 117.6583642 | 1.881686451 | 3.9E-30 | 1.15E-28 | FAM101B |
| ENSG00000273129 | 14.87173606 | 1.873680381 | 5.87E-10 | 4.28E-09 | RP5-973M2.2 |
| ENSG00000081041 | 41.49153288 | 1.86208614 | 2.23E-13 | 2.25E-12 | CXCL2 |
| ENSG00000171016 | 16.04051274 | 1.861129835 | 7.21E-10 | 5.22E-09 | PYGO1 |
| ENSG00000107954 | 212.4631498 | 1.860636023 | 2.17E-47 | 1.5E-45 | NEURL1 |
| ENSG00000138829 | 60.82803479 | 1.859533218 | 5.21E-18 | 7.5E-17 | FBN2 |
| ENSG00000162975 | 56.31920176 | 1.844067969 | 4.62E-17 | 6.17E-16 | KCNF1 |
| ENSG00000153956 | 16.85313736 | 1.831340132 | 8.76E-10 | 6.29E-09 | CACNA2D1 |
| ENSG00000197106 | 40.5295764 | 1.800002581 | 3.42E-13 | 3.4E-12 | SLC6A17 |
| ENSG00000174640 | 20.68762971 | 1.786533539 | 6E-10 | 4.38E-09 | SLCO2A1 |
| ENSG00000120337 | 2054.488217 | 1.780184457 | 3.17E-241 | 3.88E-238 | TNFSF18 |
| ENSG00000165300 | 109.4298683 | 1.773735697 | 3.22E-25 | 7.29E-24 | SLITRK5 |
| ENSG00000147852 | 19.2050662 | 1.772911343 | 1.46E-09 | 1.03E-08 | VLDLR |
| ENSG00000113356 | 294.9654116 | 1.76596991 | 1.35E-56 | 1.2E-54 | POLR3G |
| ENSG00000104369 | 289.8111074 | 1.749456306 | 1.19E-56 | 1.07E-54 | JPH1 |
| ENSG00000113389 | 154.5895694 | 1.728848166 | 4.68E-33 | 1.63E-31 | NPR3 |
| ENSG00000184613 | 92.11638758 | 1.72116938 | 3.11E-21 | 5.47E-20 | NELL2 |
| ENSG00000145147 | 289.2167829 | 1.720455046 | 1.63E-46 | 1.07E-44 | SLIT2 |
| ENSG00000221866 | 28.65877355 | 1.712811693 | 6.69E-10 | 4.86E-09 | PLXNA4 |
| ENSG00000124882 | 2904.859273 | 1.709738167 | 1.48E-242 | 2.09E-239 | EREG |
| ENSG00000163827 | 8.857313241 | 1.696084015 | 3.04E-08 | 1.85E-07 | LRRC2 |
| ENSG00000087494 | 908.4531831 | 1.694695304 | 4.93E-130 | 2.1E-127 | PTHLH |
| ENSG00000136244 | 19.53418431 | 1.690512342 | 6.72E-09 | 4.39E-08 | IL6 |
| ENSG00000112394 | 18.6844344 | 1.683642263 | 9.61E-09 | 6.17E-08 | SLC16A10 |
| ENSG00000183696 | 1455.544837 | 1.680964465 | 1.45E-174 | 1.16E-171 | UPP1 |
| ENSG00000066468 | 24.84561571 | 1.680706218 | 1.35E-09 | 9.55E-09 | FGFR2 |
| ENSG00000234964 | 19.18103294 | 1.679034738 | 8.14E-09 | 5.27E-08 | FABP5P7 |
| ENSG00000142178 | 2212.690072 | 1.666235647 | 2.89E-244 | 4.41E-241 | SIK1 |
| ENSG00000176170 | 375.8739241 | 1.665038486 | 8.05E-63 | 9.28E-61 | SPHK1 |
| ENSG00000251127 | 30.33606199 | 1.654037845 | 2.62E-10 | 1.99E-09 | RP11-280G9.1 |
| ENSG00000164362 | 14.02782704 | 1.6532211 | 6.10E-08 | 3.57E-07 | TERT |
| ENSG00000164949 | 1037.840133 | 1.644838194 | 3.67E-143 | 1.98E-140 | GEM |
| ENSG00000257042 | 22.68048232 | 1.632914944 | 6.98E-09 | 4.55E-08 | RP11-993B23.3 |
| ENSG00000073756 | 922.9008304 | 1.627214383 | 4.18E-115 | 1.44E-112 | PTGS2 |
| ENSG00000163545 | 402.8563091 | 1.625745001 | 1.88E-59 | 1.91E-57 | NUAK2 |
| ENSG00000134769 | 46.81812105 | 1.623684927 | 9.19E-12 | 7.96E-11 | DTNA |
| ENSG00000205420 | 1119.828008 | 1.620003346 | 1.52E-115 | 5.34E-113 | KRT6A |
| ENSG00000198093 | 9.184916667 | 1.614990981 | 1.36E-07 | 7.68E-07 | ZNF649 |
| ENSG00000118523 | 4459.516314 | 1.613018219 | 5E-270 | 1.02E-266 | CTGF |
| ENSG00000003096 | 96.42416102 | 1.611005968 | 6.32E-20 | 1.03E-18 | KLHL13 |
| ENSG00000163734 | 57.09468505 | 1.599067399 | 1.61E-12 | 1.49E-11 | CXCL3 |
| ENSG00000171488 | 357.9039694 | 1.593558222 | 3.53E-55 | 3.08E-53 | LRRC8C |
| ENSG00000169860 | 50.48303774 | 1.59325754 | 1.12E-12 | 1.06E-11 | P2RY1 |
| ENSG00000106366 | 634.587662 | 1.59280198 | 2.03E-92 | 4.59E-90 | SERPINE1 |
| ENSG00000175556 | 49.62514446 | 1.582906691 | 2.7E-12 | 2.46E-11 | LONRF3 |
| ENSG00000130881 | 130.2846442 | 1.58215888 | 1.1E-21 | 2E-20 | LRP3 |
| ENSG00000165449 | 7.188891303 | 1.571853432 | 1.99E-07 | 1.10E-06 | SLC16A9 |
| ENSG00000164241 | 27.15442593 | 1.568470669 | 1.57E-08 | 9.80E-08 | C5orf63 |
| ENSG00000135604 | 28.98684593 | 1.558250634 | 6.04E-09 | 3.96E-08 | STX11 |
| ENSG00000205595 | 51.95787354 | 1.552359362 | 2.94E-12 | 2.66E-11 | AREGB |
| ENSG00000107719 | 333.4498298 | 1.552155381 | 4.47E-53 | 3.67E-51 | PALD1 |
| ENSG00000226887 | 29.67090972 | 1.549544255 | 3.16E-09 | 2.15E-08 | ERVMER34-1 |
| ENSG00000118503 | 545.610429 | 1.547381201 | 2.27E-76 | 3.53E-74 | TNFAIP3 |
| ENSG00000144821 | 39.7898274 | 1.546059071 | 3.16E-10 | 2.38E-09 | MYH15 |
| ENSG00000116667 | 194.3761565 | 1.53980865 | 6.88E-31 | 2.14E-29 | C1orf21 |
| ENSG00000115457 | 179.470292 | 1.538013397 | 2.06E-29 | 5.82E-28 | IGFBP2 |
| ENSG00000121966 | 16.00661585 | 1.536229434 | 2.43E-07 | 1.32E-06 | CXCR4 |
| ENSG00000255414 | 24.83385221 | 1.531245832 | 2.09E-08 | 1.29E-07 | LINC01059 |
| ENSG00000163453 | 102.5336914 | 1.530853072 | 7.04E-19 | 1.08E-17 | IGFBP7 |
| ENSG00000102271 | 64.43455761 | 1.50867811 | 8.7E-13 | 8.34E-12 | KLHL4 |
| ENSG00000067082 | 7258.175881 | 1.506951029 | 1.99E-291 | 5.21E-288 | KLF6 |
| ENSG00000114450 | 29.67464532 | 1.506364848 | 2.12E-08 | 1.30E-07 | GNB4 |
| ENSG00000153162 | 62.65259034 | 1.504826462 | 4.61E-13 | 4.52E-12 | BMP6 |
| ENSG00000214318 | 22.84581773 | 1.500902158 | 9.84E-08 | 5.64E-07 | ATP5G1P6 |
| ENSG00000128011 | 80.60931366 | 1.49624014 | 2.67E-14 | 2.93E-13 | LRFN1 |
| ENSG00000111666 | 69.41455107 | 1.476282491 | 7.03E-14 | 7.46E-13 | CHPT1 |
| ENSG00000172602 | 80.54988783 | 1.469129764 | 7.5E-15 | 8.59E-14 | RND1 |
| ENSG00000172318 | 12.33925545 | 1.463860185 | 1.69E-06 | 8.29E-06 | B3GALT1 |
| ENSG00000136098 | 70.59193841 | 1.461778469 | 4.87E-14 | 5.24E-13 | NEK3 |
| ENSG00000171246 | 130.9900086 | 1.459087867 | 2.99E-20 | 5E-19 | NPTX1 |
| ENSG00000128050 | 3113.356519 | 1.456885441 | 5.99E-220 | 6.86E-217 | PAICS |
| ENSG00000081051 | 7.695201337 | 1.455133018 | 1.81E-06 | 8.79E-06 | AFP |
| ENSG00000131016 | 865.9865625 | 1.453649978 | 1.84E-101 | 4.88E-99 | AKAP12 |
| ENSG00000149256 | 20.67604896 | 1.452856737 | 3.41E-07 | 1.82E-06 | TENM4 |
| ENSG00000165905 | 112.5688658 | 1.446322065 | 1.03E-18 | 1.56E-17 | GYLTL1B |
| ENSG00000100505 | 32.14048633 | 1.44465044 | 1.58E-08 | 9.87E-08 | TRIM9 |
| ENSG00000166165 | 518.5282184 | 1.439861703 | 3.75E-61 | 3.99E-59 | CKB |
| ENSG00000117115 | 264.7479354 | 1.429733568 | 3.27E-38 | 1.47E-36 | PADI2 |
| ENSG00000175592 | 3841.124468 | 1.429723096 | 2.98E-242 | 3.91E-239 | FOSL1 |
| ENSG00000150995 | 52.57897713 | 1.424243866 | 1.28E-10 | 1E-09 | ITPR1 |
| ENSG00000179111 | 48.77471393 | 1.419640657 | 3.68E-10 | 2.74E-09 | HES7 |
| ENSG00000102098 | 68.56054145 | 1.419485843 | 9.23E-13 | 8.8E-12 | SCML2 |
| ENSG00000132429 | 17.66546817 | 1.413488333 | 1.32E-06 | 6.55E-06 | POPDC3 |
| ENSG00000122641 | 296.2497385 | 1.412013908 | 7.84E-37 | 3.3E-35 | INHBA |
| ENSG00000091129 | 182.3677128 | 1.40615738 | 3.23E-27 | 8.2E-26 | NRCAM |
| ENSG00000033867 | 1321.846863 | 1.404611624 | 2.33E-117 | 8.53E-115 | SLC4A7 |
| ENSG00000203867 | 131.0636024 | 1.404310985 | 8.13E-19 | 1.24E-17 | RBM20 |
| ENSG00000169469 | 137.9717819 | 1.402346703 | 2.29E-18 | 3.36E-17 | SPRR1B |
| ENSG00000132326 | 652.41877 | 1.401043055 | 4.22E-75 | 6.39E-73 | PER2 |
| ENSG00000167772 | 153.9945999 | 1.399442988 | 2.92E-21 | 5.14E-20 | ANGPTL4 |
| ENSG00000239948 | 27.99713653 | 1.399296433 | 1.78E-07 | 9.86E-07 | RN7SL368P |
| ENSG00000169884 | 79.61739804 | 1.386824177 | 2.49E-12 | 2.27E-11 | WNT10B |
| ENSG00000273108 | 18.34973686 | 1.386400206 | 3.13E-06 | 1.47E-05 | RP11-416N2.4 |
| ENSG00000225057 | 25.98665904 | 1.379118078 | 2.58E-07 | 1.40E-06 | AC096574.4 |
| ENSG00000266553 | 7.683583545 | 1.36547879 | 7.67E-06 | 3.39E-05 | RN7SL356P |
| ENSG00000155011 | 8.357076264 | 1.358882603 | 9.13E-06 | 3.98E-05 | DKK2 |
| ENSG00000109321 | 1391.198123 | 1.351131559 | 2.46E-14 | 2.69E-13 | AREG |
| ENSG00000143753 | 296.8095429 | 1.341790591 | 2.7E-31 | 8.57E-30 | DEGS1 |
| ENSG00000173391 | 19.65078459 | 1.33753294 | 2.92E-06 | 1.38E-05 | OLR1 |
| ENSG00000169515 | 6.84870076 | 1.337388836 | 9.95E-06 | 4.31E-05 | CCDC8 |
| ENSG00000157168 | 147.2269998 | 1.334071354 | 1.97E-19 | 3.11E-18 | NRG1 |
| ENSG00000100473 | 18.00517361 | 1.33169864 | 6.91E-06 | 3.07E-05 | COCH |
| ENSG00000147118 | 26.66141465 | 1.331351627 | 7.09E-07 | 3.64E-06 | ZNF182 |
| ENSG00000225614 | 214.80803 | 1.325841164 | 7.36E-28 | 1.93E-26 | ZNF469 |
| ENSG00000201302 | 17.66614427 | 1.325711277 | 4.98E-06 | 2.27E-05 | SNORA65 |
| ENSG00000136982 | 443.6964557 | 1.315799701 | 4.75E-45 | 2.89E-43 | DSCC1 |
| ENSG00000125454 | 173.5258444 | 1.312842534 | 4.84E-22 | 8.89E-21 | SLC25A19 |
| ENSG00000164105 | 46.126925 | 1.310606044 | 5.86E-09 | 3.86E-08 | SAP30 |
| ENSG00000172671 | 7.703374924 | 1.310403451 | 1.30E-05 | 5.54E-05 | ZFAND4 |
| ENSG00000145220 | 646.2722268 | 1.309261905 | 2.41E-55 | 2.12E-53 | LYAR |
| ENSG00000137273 | 80.04026346 | 1.308097717 | 2.89E-12 | 2.62E-11 | FOXF2 |
| ENSG00000176406 | 123.6229853 | 1.30503742 | 4.52E-16 | 5.65E-15 | RIMS2 |
| ENSG00000134569 | 456.9358464 | 1.303278761 | 1.48E-42 | 8.06E-41 | LRP4 |
| ENSG00000230439 | 1353.930984 | 1.301620198 | 1.08E-104 | 3.01E-102 | RP11-488P3.1 |
| ENSG00000134107 | 4377.473429 | 1.299821214 | 7.09E-214 | 7.64E-211 | BHLHE40 |
| ENSG00000158321 | 271.1539582 | 1.297557927 | 1.37E-30 | 4.16E-29 | AUTS2 |
| ENSG00000143867 | 7.180341336 | 1.294172045 | 2.08E-05 | 8.61E-05 | OSR1 |
| ENSG00000161551 | 5.351996516 | 1.286834429 | 1.17E-05 | 5.00E-05 | ZNF577 |
| ENSG00000137440 | 8963.336086 | 1.282259326 | 1.53E-253 | 2.81E-250 | FGFBP1 |
| ENSG00000120658 | 11.17967679 | 1.278892621 | 3.02E-05 | 0.00012224 | ENOX1 |
| ENSG00000213694 | 271.2200382 | 1.277392061 | 2.2E-30 | 6.61E-29 | S1PR3 |
| ENSG00000163053 | 161.5028331 | 1.276851176 | 1.66E-19 | 2.64E-18 | SLC16A14 |
| ENSG00000111490 | 221.9387973 | 1.272886252 | 7.93E-27 | 1.97E-25 | TBC1D30 |
| ENSG00000188582 | 15.50402115 | 1.272455939 | 1.84E-05 | 7.67E-05 | PAQR9 |
| ENSG00000244405 | 1164.495952 | 1.272245676 | 9.81E-99 | 2.5E-96 | ETV5 |
| ENSG00000039560 | 1343.330695 | 1.271378892 | 9.81E-103 | 2.64E-100 | RAI14 |
| ENSG00000227640 | 71.57591046 | 1.271162344 | 2.03E-10 | 1.55E-09 | SOX21-AS1 |
| ENSG00000252690 | 16.32742948 | 1.26964824 | 1.97E-05 | 8.18E-05 | SCARNA15 |
| ENSG00000134986 | 15.65607359 | 1.266910744 | 2.19E-05 | 9.04E-05 | NREP |
| ENSG00000119938 | 206.682984 | 1.256721394 | 1.1E-22 | 2.11E-21 | PPP1R3C |
| ENSG00000117280 | 470.5514868 | 1.256134308 | 7.63E-37 | 3.22E-35 | RAB7L1 |
| ENSG00000116661 | 13.33921931 | 1.254371997 | 3.61E-05 | 0.0001441 | FBXO2 |
| ENSG00000138685 | 93.83797782 | 1.250969965 | 4.81E-11 | 3.9E-10 | FGF2 |
| ENSG00000109686 | 844.5767779 | 1.246216607 | 9.64E-64 | 1.16E-61 | SH3D19 |
| ENSG00000184937 | 8.186867129 | 1.245983168 | 4.10E-05 | 0.00016224 | WT1 |
| ENSG00000224877 | 648.5128285 | 1.244785936 | 5.14E-61 | 5.41E-59 | C17orf89 |
| ENSG00000185818 | 143.9466319 | 1.24114243 | 1.33E-16 | 1.72E-15 | NAT8L |
| ENSG00000272703 | 17.15398649 | 1.237326293 | 2.09E-05 | 8.65E-05 | RP11-78A19.4 |
| ENSG00000166483 | 1899.191429 | 1.234662788 | 1.88E-85 | 3.71E-83 | WEE1 |
| ENSG00000169862 | 6.867296504 | 1.23313296 | 2.76E-05 | 0.00011247 | CTNND2 |
| ENSG00000165685 | 9.512698374 | 1.232249886 | 6.12E-05 | 0.00023519 | TMEM52B |
| ENSG00000184347 | 27.47574268 | 1.230037599 | 4.72E-06 | 2.16E-05 | SLIT3 |
| ENSG00000272016 | 315.9592412 | 1.228137181 | 3.9E-32 | 1.3E-30 | RP11-215G15.5 |
| ENSG00000138166 | 4271.772737 | 1.227806319 | 9.75E-180 | 8.51E-177 | DUSP5 |
| ENSG00000128512 | 101.145476 | 1.2272326 | 1.87E-13 | 1.9E-12 | DOCK4 |
| ENSG00000069482 | 297.5948595 | 1.221495919 | 3.11E-30 | 9.26E-29 | GAL |
| ENSG00000101384 | 2846.730983 | 1.219063649 | 1.3E-128 | 5.29E-126 | JAG1 |
| ENSG00000003989 | 12.00295496 | 1.21447858 | 7.28E-05 | 0.00027621 | SLC7A2 |
| ENSG00000043039 | 434.516328 | 1.212501249 | 1.2E-37 | 5.26E-36 | BARX2 |
| ENSG00000187498 | 914.3782013 | 1.212120094 | 1.59E-76 | 2.49E-74 | COL4A1 |
| ENSG00000150627 | 234.2003534 | 1.210742574 | 1.19E-25 | 2.78E-24 | WDR17 |
| ENSG00000255248 | 25.12482105 | 1.210689803 | 7.23E-06 | 3.21E-05 | RP11-166D19.1 |
| ENSG00000157833 | 91.18168933 | 1.210520656 | 7.11E-12 | 6.22E-11 | GAREML |
| ENSG00000185760 | 81.32638156 | 1.20902506 | 1.16E-08 | 7.37E-08 | KCNQ5 |
| ENSG00000101144 | 227.9533032 | 1.206564088 | 1.46E-21 | 2.63E-20 | BMP7 |
| ENSG00000133069 | 49.62478765 | 1.20431492 | 1.48E-07 | 8.28E-07 | TMCC2 |
| ENSG00000123700 | 239.9893678 | 1.198253249 | 6.07E-23 | 1.18E-21 | KCNJ2 |
| ENSG00000176165 | 167.3534377 | 1.196972613 | 5.33E-17 | 7.08E-16 | FOXG1 |
| ENSG00000185272 | 12.50201144 | 1.195300731 | 8.24E-05 | 0.00031006 | RBM11 |
| ENSG00000222047 | 153.5198909 | 1.193649792 | 5E-15 | 5.79E-14 | C10orf55 |
| ENSG00000071539 | 1251.88256 | 1.189011197 | 6.69E-84 | 1.28E-81 | TRIP13 |
| ENSG00000125257 | 433.0421867 | 1.188834328 | 1.68E-36 | 6.94E-35 | ABCC4 |
| ENSG00000250033 | 13.9943155 | 1.188251246 | 7.41E-05 | 0.00028091 | SLC7A11-AS1 |
| ENSG00000145416 | 59.19912433 | 1.184583991 | 1.04E-08 | 6.68E-08 | MARCHF1 |
| ENSG00000151014 | 167.2608329 | 1.183509905 | 7.85E-18 | 1.11E-16 | CCRN4L |
| ENSG00000214772 | 6.024231008 | 1.178779628 | 7.69E-05 | 0.00029088 | RP11-174G6.1 |
| ENSG00000117322 | 11.17109658 | 1.17422266 | 0.0001221 | 0.00044861 | CR2 |
| ENSG00000164045 | 487.852184 | 1.172927401 | 3.47E-45 | 2.14E-43 | CDC25A |
| ENSG00000173258 | 11.83040695 | 1.171263848 | 0.0001243 | 0.00045614 | ZNF483 |
| ENSG00000186205 | 374.8825226 | 1.170234 | 3.4E-35 | 1.33E-33 | MTARC1 |
| ENSG00000059804 | 13.82434806 | 1.169610855 | 9.79E-05 | 0.00036398 | SLC2A3 |
| ENSG00000123545 | 184.132389 | 1.168011314 | 1.34E-17 | 1.86E-16 | NDUFAF4 |
| ENSG00000249992 | 41.7862817 | 1.165288378 | 1.92E-06 | 9.29E-06 | TMEM158 |
| ENSG00000260253 | 9.012232834 | 1.158973285 | 0.0001633 | 0.00058777 | RP4-676L2.1 |
| ENSG00000026025 | 93.87069845 | 1.157613503 | 4.21E-10 | 3.11E-09 | VIM |
| ENSG00000154930 | 12.8586768 | 1.156341242 | 0.0001565 | 0.00056474 | ACSS1 |
| ENSG00000130595 | 7.193485658 | 1.155048904 | 0.0001355 | 0.0004949 | TNNT3 |
| ENSG00000233608 | 78.48859063 | 1.153388523 | 4.21E-09 | 2.82E-08 | TWIST2 |
| ENSG00000184185 | 12.17654978 | 1.150598933 | 0.000157 | 0.00056639 | KCNJ12 |
| ENSG00000198142 | 782.9561244 | 1.150095632 | 3.27E-61 | 3.53E-59 | SOWAHC |
| ENSG00000269989 | 23.99833401 | 1.148921484 | 3.15E-05 | 0.00012698 | RP11-635N19.3 |
| ENSG00000164794 | 4.515383562 | 1.147714444 | 6.42E-05 | 0.00024602 | KCNV1 |
| ENSG00000139910 | 24.31964992 | 1.147621811 | 2.61E-05 | 0.00010656 | NOVA1 |
| ENSG00000140563 | 79.98134266 | 1.144407465 | 3.55E-10 | 2.65E-09 | MCTP2 |
| ENSG00000116649 | 861.8578138 | 1.143773551 | 1.42E-63 | 1.69E-61 | SRM |
| ENSG00000111450 | 164.9320984 | 1.143672971 | 4.57E-17 | 6.11E-16 | STX2 |
| ENSG00000126249 | 210.3796687 | 1.142810657 | 3.73E-20 | 6.19E-19 | PDCD2L |
| ENSG00000132130 | 243.7194793 | 1.136236909 | 8.71E-20 | 1.41E-18 | LHX1 |
| ENSG00000113448 | 1229.743212 | 1.13552923 | 1.84E-67 | 2.44E-65 | PDE4D |
| ENSG00000165490 | 241.598227 | 1.133151277 | 4.65E-23 | 9.11E-22 | C11orf82 |
| ENSG00000105926 | 489.8880253 | 1.126853371 | 7.85E-39 | 3.62E-37 | MPP6 |
| ENSG00000136997 | 4274.603058 | 1.124810497 | 3.19E-153 | 1.95E-150 | MYC |
| ENSG00000074047 | 22.64682677 | 1.124707026 | 6.05E-05 | 0.00023269 | GLI2 |
| ENSG00000116254 | 123.3804656 | 1.123999744 | 1.02E-12 | 9.65E-12 | CHD5 |
| ENSG00000174013 | 1548.482988 | 1.118371315 | 2.42E-90 | 5.15E-88 | FBXO45 |
| ENSG00000231607 | 87.48398407 | 1.115818456 | 8.1E-10 | 5.84E-09 | DLEU2 |
| ENSG00000118515 | 1671.494827 | 1.115383475 | 2.36E-71 | 3.38E-69 | SGK1 |
| ENSG00000084710 | 11.17030653 | 1.114718561 | 0.0002619 | 0.00091363 | EFR3B |
| ENSG00000125285 | 101.7479426 | 1.114288666 | 2.43E-10 | 1.85E-09 | SOX21 |
| ENSG00000272168 | 8.673625539 | 1.11220401 | 0.0002954 | 0.00102115 | CASC15 |
| ENSG00000174371 | 532.4840738 | 1.111585572 | 1.63E-37 | 7.1E-36 | EXO1 |
| ENSG00000125266 | 462.9110603 | 1.109325942 | 5.94E-35 | 2.29E-33 | EFNB2 |
| ENSG00000143476 | 900.3819551 | 1.106659505 | 1.76E-57 | 1.63E-55 | DTL |
| ENSG00000135763 | 402.522469 | 1.106213299 | 3.17E-34 | 1.18E-32 | URB2 |
| ENSG00000198176 | 2697.67531 | 1.103456398 | 1.18E-120 | 4.4E-118 | TFDP1 |
| ENSG00000183691 | 49.1139087 | 1.0992945 | 9.39E-07 | 4.74E-06 | NOG |
| ENSG00000120875 | 9386.25631 | 1.09917767 | 2.86E-170 | 1.94E-167 | DUSP4 |
| ENSG00000100364 | 1128.314917 | 1.099104367 | 9.77E-60 | 1E-57 | KIAA0930 |
| ENSG00000171522 | 1489.016696 | 1.098243418 | 1.78E-92 | 4.07E-90 | PTGER4 |
| ENSG00000123213 | 849.3386841 | 1.095167342 | 2.26E-51 | 1.78E-49 | NLN |
| ENSG00000137713 | 621.990176 | 1.09502612 | 7.49E-47 | 5.03E-45 | PPP2R1B |
| ENSG00000213707 | 13.31277217 | 1.093211592 | 0.0003441 | 0.00117656 | HMGB1P10 |
| ENSG00000187815 | 20.96324378 | 1.091206102 | 0.000158 | 0.00056958 | ZFP69 |
| ENSG00000148339 | 1795.071182 | 1.090183762 | 7.45E-97 | 1.82E-94 | SLC25A25 |
| ENSG00000142871 | 9018.818522 | 1.088893376 | 2.26E-173 | 1.59E-170 | CYR61 |
| ENSG00000133119 | 576.4985654 | 1.086310366 | 5.67E-41 | 2.89E-39 | RFC3 |
| ENSG00000113460 | 1171.203409 | 1.0823305 | 6.79E-67 | 8.82E-65 | BRIX1 |
| ENSG00000091656 | 81.00724797 | 1.079792302 | 2.62E-09 | 1.8E-08 | ZFHX4 |
| ENSG00000251196 | 94.55734711 | 1.079787481 | 9.65E-10 | 6.9E-09 | RP11-54F2.1 |
| ENSG00000149633 | 39.57711108 | 1.078373691 | 5.11E-06 | 2.33E-05 | KIAA1755 |
| ENSG00000143942 | 92.61791713 | 1.078346253 | 2.82E-10 | 2.13E-09 | CHAC2 |
| ENSG00000101665 | 165.1959006 | 1.076984467 | 3.69E-15 | 4.35E-14 | SMAD7 |
| ENSG00000102743 | 149.7236093 | 1.07474181 | 6.71E-13 | 6.49E-12 | SLC25A15 |
| ENSG00000112759 | 979.1508816 | 1.074659567 | 1.28E-61 | 1.4E-59 | SLC29A1 |
| ENSG00000173894 | 420.9506298 | 1.073941156 | 2.71E-33 | 9.54E-32 | CBX2 |
| ENSG00000164338 | 435.9658104 | 1.073578938 | 3.34E-34 | 1.24E-32 | UTP15 |
| ENSG00000119630 | 119.7532359 | 1.072584631 | 1.37E-11 | 1.17E-10 | PGF |
| ENSG00000179094 | 493.4915044 | 1.06781703 | 6.1E-34 | 2.23E-32 | PER1 |
| ENSG00000118513 | 75.20432522 | 1.066876969 | 1.03E-07 | 5.89E-07 | MYB |
| ENSG00000010310 | 10.98674097 | 1.066824196 | 0.0004987 | 0.00165955 | GIPR |
| ENSG00000168502 | 1339.852854 | 1.0628483 | 2.76E-68 | 3.77E-66 | SOGA2 |
| ENSG00000148459 | 130.0491192 | 1.06155626 | 2.08E-12 | 1.91E-11 | PDSS1 |
| ENSG00000164251 | 1905.163432 | 1.061527041 | 2.6E-87 | 5.29E-85 | F2RL1 |
| ENSG00000144485 | 49.06760967 | 1.061112603 | 2.32E-06 | 1.11E-05 | HES6 |
| ENSG00000188042 | 1468.644718 | 1.059561581 | 1.7E-61 | 1.84E-59 | ARL4C |
| ENSG00000115946 | 534.8552129 | 1.057233312 | 2.22E-28 | 5.98E-27 | PNO1 |
| ENSG00000083635 | 186.5518691 | 1.054261406 | 1.47E-16 | 1.9E-15 | NUFIP1 |
| ENSG00000157315 | 8.850472985 | 1.054134011 | 0.0006044 | 0.00198169 | TMED6 |
| ENSG00000165572 | 320.0962289 | 1.053913977 | 2.49E-24 | 5.31E-23 | KBTBD6 |
| ENSG00000188677 | 244.2709906 | 1.048576531 | 1.11E-19 | 1.79E-18 | PARVB |
| ENSG00000121653 | 264.911943 | 1.045710222 | 7.4E-20 | 1.2E-18 | MAPK8IP1 |
| ENSG00000147224 | 885.6530664 | 1.045137706 | 2.26E-53 | 1.88E-51 | PRPS1 |
| ENSG00000184304 | 8.838752577 | 1.045105345 | 0.0006711 | 0.00217942 | PRKD1 |
| ENSG00000115008 | 661.2312052 | 1.045089723 | 2.12E-35 | 8.4E-34 | IL1A |
| ENSG00000213397 | 39.27300293 | 1.043642164 | 1.97E-05 | 8.19E-05 | HAUS7 |
| ENSG00000166831 | 11.1499033 | 1.043375861 | 0.0006393 | 0.00208601 | RBPMS2 |
| ENSG00000087470 | 1865.914354 | 1.043017466 | 2.33E-15 | 2.79E-14 | DNM1L |
| ENSG00000165810 | 74.32632799 | 1.042509655 | 7.71E-08 | 4.46E-07 | BTNL9 |
| ENSG00000144354 | 733.0742903 | 1.041063919 | 4.56E-43 | 2.55E-41 | CDCA7 |
| ENSG00000140807 | 9.002734478 | 1.041052059 | 0.0006979 | 0.00225845 | NKD1 |
| ENSG00000247626 | 726.4704466 | 1.039552634 | 6.08E-43 | 3.36E-41 | MARS2 |
| ENSG00000131650 | 137.1097368 | 1.037589368 | 4.1E-12 | 3.65E-11 | KREMEN2 |
| ENSG00000139737 | 15.97833056 | 1.034885375 | 0.0004113 | 0.00138896 | SLAIN1 |
| ENSG00000138035 | 607.4795553 | 1.032754008 | 3.98E-39 | 1.87E-37 | PNPT1 |
| ENSG00000065150 | 3635.531319 | 1.032157388 | 4.9E-106 | 1.42E-103 | IPO5 |
| ENSG00000207652 | 41.38987572 | 1.02961361 | 8.13E-06 | 3.57E-05 | MIR621 |
| ENSG00000224837 | 147.5928072 | 1.028757593 | 1.42E-12 | 1.33E-11 | GCSHP5 |
| ENSG00000166450 | 233.3881328 | 1.028007023 | 6.1E-18 | 8.74E-17 | PRTG |
| ENSG00000171243 | 8.672505069 | 1.027492064 | 0.0008248 | 0.00262953 | SOSTDC1 |
| ENSG00000181026 | 747.6278063 | 1.027188386 | 2.88E-46 | 1.85E-44 | AEN |
| ENSG00000117118 | 2198.407934 | 1.022423521 | 6.37E-80 | 1.08E-77 | SDHB |
| ENSG00000232874 | 24.10301213 | 1.021797336 | 0.0002 | 0.00071033 | RP11-135A1.2 |
| ENSG00000127920 | 145.9742959 | 1.021169429 | 2.35E-11 | 1.95E-10 | GNG11 |
| ENSG00000136014 | 8.013143491 | 1.020876186 | 0.0008779 | 0.00278472 | USP44 |
| ENSG00000133107 | 19.30759698 | 1.018145239 | 0.00045 | 0.00150893 | TRPC4 |
| ENSG00000198720 | 274.0295659 | 1.01666838 | 1.09E-19 | 1.75E-18 | ANKRD13B |
| ENSG00000141519 | 54.38083014 | 1.015937353 | 2.75E-06 | 1.30E-05 | CCDC40 |
| ENSG00000180801 | 449.9458063 | 1.015195952 | 5.27E-32 | 1.75E-30 | ARSJ |
| ENSG00000167552 | 156.2961518 | 1.014078184 | 2.23E-11 | 1.86E-10 | TUBA1A |
| ENSG00000030066 | 2551.802829 | 1.013376104 | 7.22E-108 | 2.24E-105 | NUP160 |
| ENSG00000119929 | 353.9607115 | 1.012563082 | 1.52E-24 | 3.3E-23 | CUTC |
| ENSG00000111845 | 364.1137596 | 1.012266166 | 9.03E-26 | 2.13E-24 | PAK1IP1 |
| ENSG00000188820 | 8.330698308 | 1.010199053 | 0.0010129 | 0.0031683 | FAM26F |
| ENSG00000136111 | 974.8459493 | 1.007956131 | 8.08E-50 | 5.97E-48 | TBC1D4 |
| ENSG00000084674 | 5.845973154 | 1.00639415 | 0.0007976 | 0.0025491 | APOB |
| ENSG00000107130 | 1098.742189 | 1.002820541 | 8.55E-52 | 6.81E-50 | NCS1 |
| ENSG00000123358 | 7001.419119 | 1.001826428 | 1.83E-135 | 8.39E-133 | NR4A1 |
| ENSG00000189410 | 32.25013119 | 1.001396604 | 9.20E-05 | 0.00034331 | SH2D5 |
| ENSG00000262877 | 31.88671976 | -1.00057727 | 0.0001023 | 0.00037973 | RP11-1055B8.4 |
| ENSG00000250072 | 34.48086443 | -1.001147833 | 4.25E-05 | 0.00016767 | CTC-529P8.1 |
| ENSG00000168517 | 44.21357898 | -1.001428079 | 1.27E-05 | 5.44E-05 | HEXIM2 |
| ENSG00000223813 | 67.95521421 | -1.001688231 | 4.56E-07 | 2.40E-06 | AC007255.8 |
| ENSG00000214357 | 165.4549267 | -1.001908106 | 2.1E-13 | 2.13E-12 | NEURL1B |
| ENSG00000203306 | 447.917312 | -1.001987366 | 3.97E-28 | 1.06E-26 | AP001007.1 |
| ENSG00000128394 | 146.3148195 | -1.00278102 | 2.14E-12 | 1.97E-11 | APOBEC3F |
| ENSG00000197774 | 489.8564282 | -1.003386359 | 1.78E-27 | 4.56E-26 | EME2 |
| ENSG00000189060 | 1614.961125 | -1.004125014 | 1.09E-58 | 1.05E-56 | H1F0 |
| ENSG00000142089 | 2190.586771 | -1.004665289 | 3.91E-84 | 7.63E-82 | IFITM3 |
| ENSG00000270115 | 29.70240631 | -1.004845946 | 0.0001014 | 0.00037639 | RP11-415J8.7 |
| ENSG00000257702 | 13.84060501 | -1.006145366 | 0.0008746 | 0.00277488 | LBX2-AS1 |
| ENSG00000164855 | 758.9888058 | -1.006146958 | 1.86E-40 | 9.26E-39 | TMEM184A |
| ENSG00000139194 | 80.36950408 | -1.006646761 | 2.09E-08 | 1.29E-07 | RBP5 |
| ENSG00000160094 | 470.2851983 | -1.006700674 | 1.03E-30 | 3.17E-29 | ZNF362 |
| ENSG00000241014 | 26.73650446 | -1.007222533 | 0.0001542 | 0.0005576 | RP11-244H3.1 |
| ENSG00000125170 | 210.3823634 | -1.007237033 | 9.86E-16 | 1.2E-14 | DOK4 |
| ENSG00000255045 | 6.595010156 | -1.009366562 | 0.000849 | 0.0026988 | RP11-677M14.2 |
| ENSG00000213928 | 68.49721295 | -1.009563336 | 1.84E-06 | 8.92E-06 | IRF9 |
| ENSG00000105559 | 43.75937869 | -1.009642894 | 8.50E-06 | 3.72E-05 | PLEKHA4 |
| ENSG00000215375 | 106.7195236 | -1.009748696 | 2.28E-09 | 1.57E-08 | MYL5 |
| ENSG00000257084 | 5.928734111 | -1.009962703 | 0.0007772 | 0.00248728 | U47924.27 |
| ENSG00000222894 | 10.55024661 | -1.010456295 | 0.000976 | 0.00306517 | AL662800.1 |
| ENSG00000135253 | 117.729521 | -1.010888311 | 1.97E-10 | 1.51E-09 | KCP |
| ENSG00000198518 | 71.13403161 | -1.01095435 | 1.34E-07 | 7.55E-07 | HIST1H4E |
| ENSG00000137959 | 10.85333343 | -1.012974199 | 0.0009662 | 0.00303865 | IFI44L |
| ENSG00000106328 | 5.926762491 | -1.013363353 | 0.0007517 | 0.00241601 | FSCN3 |
| ENSG00000174233 | 1408.564934 | -1.013824889 | 7.77E-57 | 7.01E-55 | ADCY6 |
| ENSG00000184925 | 16.48559183 | -1.014277081 | 0.0004911 | 0.00163741 | LCN12 |
| ENSG00000135114 | 416.3962413 | -1.015549254 | 4.24E-29 | 1.17E-27 | OASL |
| ENSG00000221963 | 822.0992635 | -1.017328548 | 9.59E-48 | 6.68E-46 | APOL6 |
| ENSG00000230650 | 84.79223246 | -1.018116156 | 2.69E-08 | 1.64E-07 | AC112229.1 |
| ENSG00000231274 | 33.6714824 | -1.018406203 | 5.65E-05 | 0.0002181 | SBK3 |
| ENSG00000113555 | 33.34140967 | -1.019193353 | 3.82E-05 | 0.00015192 | PCDH12 |
| ENSG00000100365 | 19.96325784 | -1.019582417 | 0.0003687 | 0.00125342 | NCF4 |
| ENSG00000000971 | 107.9533264 | -1.019659291 | 3.3E-10 | 2.47E-09 | CFH |
| ENSG00000196296 | 106.8181641 | -1.019730649 | 2.22E-10 | 1.68E-09 | ATP2A1 |
| ENSG00000262903 | 20.3051795 | -1.020005908 | 0.0003118 | 0.0010744 | RP11-235E17.6 |
| ENSG00000237886 | 74.11046226 | -1.021110963 | 7.94E-08 | 4.59E-07 | RP11-611D20.2 |
| ENSG00000270021 | 15.35045456 | -1.021243836 | 0.000713 | 0.00230176 | CTC-203F4.2 |
| ENSG00000213462 | 634.714448 | -1.021635293 | 2.79E-37 | 1.2E-35 | ERV3-1 |
| ENSG00000272796 | 71.94203338 | -1.021841853 | 6.69E-08 | 3.90E-07 | RP1-74M1.3 |
| ENSG00000163472 | 392.7571756 | -1.023752548 | 3.67E-27 | 9.24E-26 | TMEM79 |
| ENSG00000254614 | 94.55899496 | -1.02381388 | 3.54E-09 | 2.4E-08 | AP003068.23 |
| ENSG00000134716 | 147.5622889 | -1.023840676 | 1.5E-13 | 1.54E-12 | CYP2J2 |
| ENSG00000267163 | 8.56154378 | -1.024350911 | 0.0008616 | 0.00273686 | AC084219.3 |
| ENSG00000181355 | 10.51464003 | -1.025600631 | 0.0008457 | 0.0026906 | OFCC1 |
| ENSG00000151303 | 8.370315988 | -1.026185616 | 0.0007533 | 0.00241966 | AGAP11 |
| ENSG00000165390 | 339.7894446 | -1.026614024 | 1.89E-05 | 7.89E-05 | ANXA8 |
| ENSG00000197558 | 18.3010596 | -1.028068354 | 0.0003523 | 0.00120242 | SSPO |
| ENSG00000173467 | 19.30473862 | -1.028070165 | 0.0003023 | 0.0010436 | AGR3 |
| ENSG00000178685 | 216.9581027 | -1.029784241 | 8.11E-18 | 1.15E-16 | PARP10 |
| ENSG00000117226 | 474.8336622 | -1.029848521 | 6.52E-33 | 2.25E-31 | GBP3 |
| ENSG00000164463 | 222.8643325 | -1.029894331 | 3.52E-17 | 4.75E-16 | CREBRF |
| ENSG00000229689 | 133.3700308 | -1.029912999 | 2.35E-12 | 2.14E-11 | AC009237.8 |
| ENSG00000248905 | 291.6051861 | -1.030226422 | 2.27E-21 | 4.03E-20 | FMN1 |
| ENSG00000198246 | 642.7501593 | -1.031059222 | 7.12E-42 | 3.76E-40 | SLC29A3 |
| ENSG00000106123 | 138.3265189 | -1.032042355 | 3.28E-12 | 2.95E-11 | EPHB6 |
| ENSG00000164308 | 1253.978082 | -1.032354116 | 1.36E-64 | 1.67E-62 | ERAP2 |
| ENSG00000235109 | 246.2530548 | -1.032432046 | 7.14E-18 | 1.02E-16 | ZSCAN31 |
| ENSG00000148357 | 6.583325723 | -1.032453085 | 0.000669 | 0.00217329 | HMCN2 |
| ENSG00000095321 | 18.9569211 | -1.03278468 | 0.0003219 | 0.00110687 | CRAT |
| ENSG00000249413 | 26.71806639 | -1.03409183 | 0.000112 | 0.00041348 | RP11-25H12.1 |
| ENSG00000138835 | 327.1797534 | -1.034550945 | 3.67E-25 | 8.28E-24 | RGS3 |
| ENSG00000130829 | 42.74264616 | -1.036326896 | 4.82E-06 | 2.20E-05 | DUSP9 |
| ENSG00000211452 | 8.398072749 | -1.036448611 | 0.0007293 | 0.00234945 | DIO1 |
| ENSG00000129038 | 27.72024681 | -1.036994491 | 8.55E-05 | 0.0003207 | LOXL1 |
| ENSG00000170581 | 1159.440028 | -1.037517474 | 8.65E-68 | 1.17E-65 | STAT2 |
| ENSG00000135407 | 89.59496875 | -1.038278613 | 3.8E-09 | 2.57E-08 | AVIL |
| ENSG00000214960 | 71.6282436 | -1.038541566 | 5.49E-08 | 3.24E-07 | ISPD |
| ENSG00000143850 | 1050.862015 | -1.038777798 | 5.84E-39 | 2.72E-37 | PLEKHA6 |
| ENSG00000130827 | 908.8633705 | -1.039086651 | 1.34E-46 | 8.9E-45 | PLXNA3 |
| ENSG00000111801 | 83.84494362 | -1.040192334 | 4.66E-09 | 3.11E-08 | BTN3A3 |
| ENSG00000185924 | 18.77759568 | -1.040473792 | 0.0003412 | 0.00116801 | RTN4RL1 |
| ENSG00000142765 | 476.7140956 | -1.040592934 | 5.87E-34 | 2.15E-32 | SYTL1 |
| ENSG00000204021 | 13.13592987 | -1.040698069 | 0.0007045 | 0.00227711 | LIPK |
| ENSG00000186526 | 8.880483847 | -1.041067115 | 0.0007055 | 0.00227955 | CYP4F8 |
| ENSG00000128298 | 64.54090181 | -1.041302978 | 1.57E-07 | 8.76E-07 | BAIAP2L2 |
| ENSG00000117400 | 10.3833492 | -1.043698128 | 0.0006723 | 0.00218283 | MPL |
| ENSG00000184995 | 29.70591142 | -1.043717389 | 4.61E-05 | 0.00018085 | IFNE |
| ENSG00000137868 | 1089.959624 | -1.044424109 | 7.3E-59 | 7.12E-57 | STRA6 |
| ENSG00000258539 | 10.34608734 | -1.046275723 | 0.0006669 | 0.00216726 | RP11-12J10.3 |
| ENSG00000154035 | 528.4668827 | -1.046523853 | 7.15E-34 | 2.59E-32 | C17orf103 |
| ENSG00000166535 | 35.93261071 | -1.046527452 | 1.65E-05 | 6.92E-05 | A2ML1 |
| ENSG00000254815 | 16.97842537 | -1.047654972 | 0.0003227 | 0.00110929 | RP11-496I9.1 |
| ENSG00000078804 | 416.6417407 | -1.048104941 | 3.46E-31 | 1.09E-29 | TP53INP2 |
| ENSG00000111405 | 15.67680066 | -1.048480449 | 0.0004209 | 0.00141857 | ENDOU |
| ENSG00000106066 | 56.92305932 | -1.048853065 | 3.23E-07 | 1.73E-06 | CPVL |
| ENSG00000264187 | 30.06728914 | -1.050571608 | 8.32E-05 | 0.00031249 | RP11-45M22.4 |
| ENSG00000107618 | 8.23469311 | -1.05070232 | 0.0006256 | 0.00204551 | RBP3 |
| ENSG00000123384 | 2753.120214 | -1.051945707 | 2.02E-54 | 1.72E-52 | LRP1 |
| ENSG00000203799 | 51.52149923 | -1.052154546 | 5.96E-06 | 2.68E-05 | CCDC162P |
| ENSG00000232352 | 5.096165177 | -1.052756901 | 0.0003527 | 0.00120362 | SEMA3B-AS1 |
| ENSG00000261786 | 29.54768251 | -1.052929134 | 5.14E-05 | 0.00019981 | RP4-555D20.2 |
| ENSG00000100346 | 8.576565618 | -1.052931687 | 0.0006003 | 0.00196897 | CACNA1I |
| ENSG00000172348 | 5.914714567 | -1.053505515 | 0.000403 | 0.00136164 | RCAN2 |
| ENSG00000121380 | 7.730458206 | -1.055237148 | 0.0005788 | 0.0019056 | BCL2L14 |
| ENSG00000173193 | 1049.901414 | -1.057492574 | 6.05E-46 | 3.84E-44 | PARP14 |
| ENSG00000216331 | 14.17294604 | -1.058242503 | 0.0004295 | 0.0014456 | HIST1H1PS1 |
| ENSG00000260265 | 137.7440261 | -1.05887841 | 3.07E-12 | 2.77E-11 | RP11-44F21.5 |
| ENSG00000149809 | 149.7358637 | -1.059022639 | 3.36E-14 | 3.65E-13 | TM7SF2 |
| ENSG00000164764 | 10.51937181 | -1.059141494 | 0.0005565 | 0.00183586 | SBSPON |
| ENSG00000008086 | 48.49173834 | -1.060080768 | 1.17E-06 | 5.82E-06 | CDKL5 |
| ENSG00000262979 | 18.15860528 | -1.060085269 | 0.0003098 | 0.00106802 | CTD-2047H16.2 |
| ENSG00000163565 | 71.54911982 | -1.060106135 | 1.87E-07 | 1.03E-06 | IFI16 |
| ENSG00000100031 | 78.71691367 | -1.060588286 | 1.22E-08 | 7.73E-08 | GGT1 |
| ENSG00000187994 | 156.1534166 | -1.060890709 | 1.91E-14 | 2.12E-13 | RINL |
| ENSG00000272944 | 7.056585202 | -1.061801085 | 0.0004621 | 0.00154615 | CTD-2308L22.1 |
| ENSG00000089127 | 791.133312 | -1.062717054 | 4.25E-45 | 2.61E-43 | OAS1 |
| ENSG00000178226 | 28.54974669 | -1.062991878 | 4.45E-05 | 0.00017494 | PRSS36 |
| ENSG00000241322 | 66.91259653 | -1.063345385 | 2.76E-07 | 1.49E-06 | CDRT1 |
| ENSG00000239779 | 243.4526347 | -1.064373194 | 8.93E-19 | 1.36E-17 | WBP1 |
| ENSG00000155066 | 3908.305794 | -1.064408619 | 6.14E-104 | 1.68E-101 | PROM2 |
| ENSG00000231890 | 21.26453512 | -1.064696831 | 0.0001335 | 0.00048846 | AC093391.2 |
| ENSG00000266964 | 5.746144644 | -1.064730897 | 0.0003676 | 0.00125014 | FXYD1 |
| ENSG00000205426 | 47.35273605 | -1.064737864 | 2.24E-06 | 1.08E-05 | KRT81 |
| ENSG00000064205 | 16.68796131 | -1.066632407 | 0.0003361 | 0.00115135 | WISP2 |
| ENSG00000258130 | 12.02416071 | -1.066702299 | 0.0004509 | 0.00151144 | RP11-347C12.3 |
| ENSG00000215045 | 26.58267315 | -1.068775496 | 9.04E-05 | 0.00033772 | GRID2IP |
| ENSG00000138642 | 192.3338356 | -1.071434355 | 1.82E-15 | 2.18E-14 | HERC6 |
| ENSG00000225969 | 49.8505938 | -1.071851537 | 6.85E-07 | 3.52E-06 | LINC00035 |
| ENSG00000134827 | 41.39481721 | -1.072320032 | 6.11E-06 | 2.74E-05 | TCN1 |
| ENSG00000170044 | 6.059270215 | -1.072575985 | 0.000323 | 0.00111013 | ZPLD1 |
| ENSG00000254429 | 19.14573981 | -1.074011234 | 0.0002034 | 0.00072223 | CTD-2562J17.7 |
| ENSG00000100299 | 90.82672438 | -1.074411019 | 1.17E-09 | 8.33E-09 | ARSA |
| ENSG00000266037 | 10.69882803 | -1.07473552 | 0.0004594 | 0.00153833 | RN7SL3 |
| ENSG00000204397 | 11.53939636 | -1.075234572 | 0.000419 | 0.00141279 | CARD16 |
| ENSG00000187260 | 7.729574245 | -1.075879405 | 0.0004202 | 0.00141639 | WDR86 |
| ENSG00000174938 | 749.8550915 | -1.075956501 | 2.46E-47 | 1.69E-45 | SEZ6L2 |
| ENSG00000160951 | 7.603712726 | -1.076780683 | 0.0003708 | 0.00126015 | PTGER1 |
| ENSG00000204420 | 41.85722527 | -1.077155236 | 3.98E-06 | 1.84E-05 | C6orf25 |
| ENSG00000230454 | 10.23442601 | -1.078686876 | 0.0004461 | 0.00149708 | U73166.2 |
| ENSG00000167100 | 21.78290387 | -1.078850039 | 0.0001794 | 0.00064204 | SAMD14 |
| ENSG00000260781 | 39.77287311 | -1.079030642 | 4.82E-06 | 2.20E-05 | ARHGAP23P1 |
| ENSG00000169116 | 40.93947231 | -1.079723266 | 4.60E-06 | 2.11E-05 | PARM1 |
| ENSG00000169435 | 92.4515975 | -1.079992226 | 4.01E-10 | 2.97E-09 | RASSF6 |
| ENSG00000263934 | 156.0709627 | -1.082104909 | 3.45E-12 | 3.09E-11 | SNORD3A |
| ENSG00000158125 | 355.2803147 | -1.082624807 | 9.09E-29 | 2.49E-27 | XDH |
| ENSG00000163898 | 2240.115868 | -1.083313203 | 6.9E-106 | 1.98E-103 | LIPH |
| ENSG00000157502 | 26.19103989 | -1.08388043 | 7.07E-05 | 0.0002692 | MUM1L1 |
| ENSG00000140623 | 6.912618443 | -1.084651284 | 0.0003674 | 0.00124966 | SEPTIN12 |
| ENSG00000233198 | 60.91212575 | -1.084700174 | 1.27E-07 | 7.20E-07 | RNF224 |
| ENSG00000273010 | 12.17605288 | -1.084825801 | 0.0003555 | 0.00121254 | RP11-96K19.5 |
| ENSG00000068976 | 117.8342227 | -1.084993695 | 5.11E-12 | 4.51E-11 | PYGM |
| ENSG00000161643 | 16.31213212 | -1.085371521 | 0.0002117 | 0.00074874 | SIGLEC16 |
| ENSG00000119917 | 345.4714206 | -1.085476126 | 1.25E-27 | 3.25E-26 | IFIT3 |
| ENSG00000159200 | 969.2431629 | -1.086332104 | 5.76E-63 | 6.72E-61 | RCAN1 |
| ENSG00000151882 | 26.01450864 | -1.086968578 | 0.0001218 | 0.00044754 | CCL28 |
| ENSG00000169035 | 9.721513201 | -1.088454439 | 0.0003989 | 0.00134877 | KLK7 |
| ENSG00000204970 | 9.570747356 | -1.088463201 | 0.0003972 | 0.00134373 | PCDHA1 |
| ENSG00000237515 | 38.43279805 | -1.088992401 | 5.38E-06 | 2.44E-05 | SHISA9 |
| ENSG00000147689 | 241.4798313 | -1.090601094 | 1.07E-20 | 1.83E-19 | FAM83A |
| ENSG00000167614 | 87.4726262 | -1.092585279 | 1.37E-09 | 9.63E-09 | TTYH1 |
| ENSG00000085514 | 22.77579316 | -1.092867752 | 7.42E-05 | 0.00028114 | PILRA |
| ENSG00000237807 | 6.590671215 | -1.092913593 | 0.0002978 | 0.00102922 | RP11-400K9.4 |
| ENSG00000178977 | 12.3428502 | -1.093846039 | 0.0003539 | 0.00120728 | LINC00324 |
| ENSG00000197191 | 120.8553516 | -1.096005861 | 5.02E-12 | 4.44E-11 | C9orf169 |
| ENSG00000176919 | 22.9307786 | -1.098085576 | 7.18E-05 | 0.00027297 | C8G |
| ENSG00000250685 | 7.563115234 | -1.101598199 | 0.0003194 | 0.00109892 | RP11-486L19.2 |
| ENSG00000172460 | 31.33658358 | -1.103321369 | 1.41E-05 | 5.98E-05 | PRSS30P |
| ENSG00000166669 | 16.18061216 | -1.103894505 | 0.0002308 | 0.00081185 | ATF7IP2 |
| ENSG00000224074 | 7.895507674 | -1.104159566 | 0.0003015 | 0.00104084 | LINC00691 |
| ENSG00000214193 | 402.1623447 | -1.104239069 | 2E-33 | 7.09E-32 | SH3D21 |
| ENSG00000065717 | 114.2790711 | -1.104263968 | 3.95E-11 | 3.22E-10 | TLE2 |
| ENSG00000139946 | 11.69631763 | -1.1050218 | 0.0002786 | 0.00096761 | PELI2 |
| ENSG00000255303 | 8.570529153 | -1.105893348 | 0.0003067 | 0.00105795 | OR5BA1P |
| ENSG00000249641 | 31.44102402 | -1.107104357 | 2.47E-05 | 0.00010146 | HOXC13-AS |
| ENSG00000105519 | 3086.617837 | -1.1071694 | 6.41E-105 | 1.81E-102 | CAPS |
| ENSG00000071909 | 28.69077447 | -1.107195396 | 1.92E-05 | 7.98E-05 | MYO3B |
| ENSG00000167779 | 550.7614574 | -1.107752194 | 1.5E-34 | 5.65E-33 | IGFBP6 |
| ENSG00000136457 | 41.99158704 | -1.107963415 | 4.41E-06 | 2.03E-05 | CHAD |
| ENSG00000103888 | 122.5248573 | -1.109564535 | 7.72E-11 | 6.14E-10 | KIAA1199 |
| ENSG00000264772 | 115.0222451 | -1.109904681 | 2.11E-12 | 1.94E-11 | SNORA67 |
| ENSG00000135437 | 14.1401025 | -1.112366781 | 0.000214 | 0.00075596 | RDH5 |
| ENSG00000060656 | 934.6866203 | -1.115817739 | 6.17E-69 | 8.57E-67 | PTPRU |
| ENSG00000171444 | 46.38116624 | -1.116273718 | 5.90E-07 | 3.07E-06 | MCC |
| ENSG00000074527 | 1598.760887 | -1.116399424 | 1.24E-83 | 2.35E-81 | NTN4 |
| ENSG00000176024 | 49.64827495 | -1.117058579 | 3.79E-07 | 2.01E-06 | ZNF613 |
| ENSG00000214733 | 20.64056532 | -1.117635439 | 8.29E-05 | 0.00031153 | RP11-429J17.8 |
| ENSG00000071991 | 92.07622426 | -1.11778608 | 8.83E-11 | 6.99E-10 | CDH19 |
| ENSG00000261305 | 14.67145812 | -1.118080905 | 0.0001818 | 0.00064948 | RP4-584D14.7 |
| ENSG00000176244 | 35.76839008 | -1.118523005 | 4.14E-06 | 1.91E-05 | ACBD7 |
| ENSG00000204219 | 10.85367417 | -1.118786509 | 0.0002564 | 0.00089565 | TCEA3 |
| ENSG00000198483 | 10.87407228 | -1.119127919 | 0.0002555 | 0.00089291 | ANKRD35 |
| ENSG00000234546 | 21.4420895 | -1.120677631 | 5.48E-05 | 0.00021201 | RP3-510D11.2 |
| ENSG00000182472 | 159.2134093 | -1.120793805 | 8.74E-14 | 9.17E-13 | CAPN12 |
| ENSG00000258839 | 415.7052749 | -1.121641807 | 3.63E-32 | 1.21E-30 | MC1R |
| ENSG00000107736 | 168.3345127 | -1.124409939 | 4.19E-16 | 5.27E-15 | CDH23 |
| ENSG00000164411 | 50.93693197 | -1.126081087 | 2.32E-07 | 1.27E-06 | GJB7 |
| ENSG00000076555 | 29.71612751 | -1.128163346 | 1.30E-05 | 5.53E-05 | ACACB |
| ENSG00000205885 | 299.1179628 | -1.128179837 | 1.16E-24 | 2.55E-23 | C1RL-AS1 |
| ENSG00000143369 | 413.3844541 | -1.129974709 | 4.92E-34 | 1.81E-32 | ECM1 |
| ENSG00000197846 | 29.16194053 | -1.130810791 | 3.69E-05 | 0.00014716 | HIST1H2BF |
| ENSG00000188505 | 7.404588997 | -1.131060036 | 0.0002064 | 0.00073168 | NCCRP1 |
| ENSG00000260633 | 100.8221195 | -1.131367152 | 1.48E-11 | 1.25E-10 | RP11-375I20.6 |
| ENSG00000135116 | 66.34368601 | -1.132254372 | 1.84E-08 | 1.14E-07 | HRK |
| ENSG00000112041 | 19.46674094 | -1.132959324 | 9.20E-05 | 0.00034326 | TULP1 |
| ENSG00000272091 | 24.87864206 | -1.136574588 | 2.35E-05 | 9.65E-05 | RP4-758J24.5 |
| ENSG00000143412 | 789.1295342 | -1.137369405 | 1.05E-60 | 1.1E-58 | ANXA9 |
| ENSG00000130045 | 39.59246744 | -1.137987624 | 3.95E-06 | 1.83E-05 | NXNL2 |
| ENSG00000107821 | 126.0421624 | -1.140197137 | 1.29E-13 | 1.33E-12 | KAZALD1 |
| ENSG00000199038 | 6.591351778 | -1.141705663 | 0.0001421 | 0.00051716 | MIR210 |
| ENSG00000239282 | 53.4347153 | -1.14208578 | 2.48E-07 | 1.35E-06 | GATSL3 |
| ENSG00000180573 | 840.4333157 | -1.142605109 | 3E-59 | 2.98E-57 | HIST1H2AC |
| ENSG00000169550 | 74.88328649 | -1.143131127 | 4.15E-09 | 2.79E-08 | MUC15 |
| ENSG00000140955 | 10.53385014 | -1.144835609 | 0.0001876 | 0.00066851 | ADAD2 |
| ENSG00000064042 | 770.4582238 | -1.145591418 | 1.58E-58 | 1.52E-56 | LIMCH1 |
| ENSG00000196517 | 459.5853665 | -1.147245985 | 1.84E-40 | 9.17E-39 | SLC6A9 |
| ENSG00000128422 | 61.16519748 | -1.148002725 | 1.45E-08 | 9.08E-08 | KRT17 |
| ENSG00000170962 | 117.9927263 | -1.149159288 | 4.54E-13 | 4.45E-12 | PDGFD |
| ENSG00000139178 | 560.9245345 | -1.149844855 | 6.96E-48 | 4.88E-46 | C1RL |
| ENSG00000253552 | 111.0254578 | -1.150256221 | 1.03E-11 | 8.84E-11 | HOXA-AS2 |
| ENSG00000197448 | 2114.819086 | -1.15064344 | 3.73E-80 | 6.45E-78 | GSTK1 |
| ENSG00000185432 | 289.947429 | -1.150673912 | 3.64E-25 | 8.22E-24 | METTL7A |
| ENSG00000203722 | 26.21650093 | -1.152423201 | 1.42E-05 | 6.03E-05 | RAET1G |
| ENSG00000118557 | 21.24648005 | -1.15271924 | 5.06E-05 | 0.00019678 | PMFBP1 |
| ENSG00000159588 | 87.26936519 | -1.154083345 | 4.92E-09 | 3.27E-08 | CCDC17 |
| ENSG00000169302 | 8.05069967 | -1.154619504 | 0.0001647 | 0.00059201 | STK32A |
| ENSG00000248015 | 19.44785193 | -1.154658952 | 0.0001002 | 0.00037235 | AC005329.7 |
| ENSG00000215769 | 144.4229682 | -1.154836206 | 4.99E-15 | 5.78E-14 | hsa-mir-6080 |
| ENSG00000235027 | 15.62912984 | -1.157160246 | 9.55E-05 | 0.00035568 | AC068580.6 |
| ENSG00000260733 | 4.917679502 | -1.1581233 | 5.63E-05 | 0.00021718 | RP11-264L1.4 |
| ENSG00000158055 | 386.4303513 | -1.158224643 | 4.85E-32 | 1.61E-30 | GRHL3 |
| ENSG00000231226 | 22.08728401 | -1.158472469 | 3.14E-05 | 0.00012658 | TRIM31-AS1 |
| ENSG00000114626 | 201.9655394 | -1.158973888 | 3.25E-20 | 5.42E-19 | ABTB1 |
| ENSG00000231924 | 251.5431363 | -1.160026574 | 9.13E-24 | 1.89E-22 | PSG1 |
| ENSG00000186807 | 37.24856096 | -1.163054567 | 5.77E-06 | 2.60E-05 | ANXA8L2 |
| ENSG00000205560 | 48.79718386 | -1.163188147 | 1.72E-07 | 9.55E-07 | CPT1B |
| ENSG00000185128 | 672.1491083 | -1.163319802 | 8.38E-41 | 4.24E-39 | TBC1D3F |
| ENSG00000071242 | 125.8627152 | -1.164662205 | 2.56E-14 | 2.8E-13 | RPS6KA2 |
| ENSG00000125508 | 19.78347952 | -1.165479784 | 5.24E-05 | 0.0002034 | SRMS |
| ENSG00000166596 | 20.75793632 | -1.168558216 | 3.89E-05 | 0.00015428 | WDR16 |
| ENSG00000214021 | 283.1197184 | -1.168959631 | 1.41E-26 | 3.46E-25 | TTLL3 |
| ENSG00000225032 | 83.80857091 | -1.170389494 | 5.38E-10 | 3.95E-09 | RP11-228B15.4 |
| ENSG00000175147 | 15.82111086 | -1.171355782 | 7.76E-05 | 0.00029331 | TMEM51-AS1 |
| ENSG00000006025 | 413.2646422 | -1.173221013 | 7.31E-38 | 3.24E-36 | OSBPL7 |
| ENSG00000267288 | 33.11344982 | -1.173326234 | 1.49E-05 | 6.30E-05 | RP13-890H12.2 |
| ENSG00000185522 | 40.23266816 | -1.174674712 | 1.40E-06 | 6.91E-06 | C11orf35 |
| ENSG00000197409 | 62.17666443 | -1.174715034 | 7.63E-09 | 4.94E-08 | HIST1H3D |
| ENSG00000166801 | 599.9534221 | -1.177343631 | 1.1E-45 | 6.92E-44 | FAM111A |
| ENSG00000273174 | 15.64841617 | -1.179144829 | 6.27E-05 | 0.00024026 | RP11-434H6.6 |
| ENSG00000006555 | 49.32537762 | -1.17985715 | 1.16E-07 | 6.57E-07 | TTC22 |
| ENSG00000154768 | 13.653834 | -1.181896844 | 8.71E-05 | 0.00032643 | C17orf50 |
| ENSG00000005108 | 8.060273191 | -1.182082538 | 0.0001172 | 0.00043127 | THSD7A |
| ENSG00000104361 | 332.4159709 | -1.182543292 | 1.27E-31 | 4.12E-30 | NIPAL2 |
| ENSG00000127415 | 122.7510977 | -1.18331536 | 1.24E-11 | 1.06E-10 | IDUA |
| ENSG00000267709 | 17.11303128 | -1.183819736 | 5.60E-05 | 0.00021611 | AC024592.9 |
| ENSG00000234290 | 50.17590559 | -1.188346232 | 9.36E-08 | 5.38E-07 | AC116366.6 |
| ENSG00000143502 | 16.61156659 | -1.189772843 | 5.46E-05 | 0.0002114 | SUSD4 |
| ENSG00000168427 | 19.11934614 | -1.18993042 | 3.05E-05 | 0.0001234 | KLHL30 |
| ENSG00000139899 | 18.29851662 | -1.192401206 | 4.01E-05 | 0.00015891 | CBLN3 |
| ENSG00000162069 | 1115.597832 | -1.192514393 | 2.75E-79 | 4.59E-77 | CCDC64B |
| ENSG00000185499 | 135.714258 | -1.19339057 | 2.84E-15 | 3.36E-14 | MUC1 |
| ENSG00000241527 | 13.32996368 | -1.193610298 | 8.00E-05 | 0.00030154 | CA15P1 |
| ENSG00000139192 | 199.2674971 | -1.193640325 | 6.11E-20 | 9.96E-19 | TAPBPL |
| ENSG00000162511 | 11.37362396 | -1.195073246 | 9.77E-05 | 0.00036339 | LAPTM5 |
| ENSG00000111335 | 121.1249282 | -1.195160716 | 7.81E-15 | 8.92E-14 | OAS2 |
| ENSG00000272720 | 34.31031393 | -1.195433603 | 1.80E-06 | 8.75E-06 | CTA-228A9.3 |
| ENSG00000090238 | 491.7376331 | -1.196295546 | 2.73E-39 | 1.3E-37 | YPEL3 |
| ENSG00000226200 | 37.91998971 | -1.197512556 | 5.10E-07 | 2.67E-06 | RP11-50E11.3 |
| ENSG00000204282 | 62.99322096 | -1.198626354 | 3.45E-09 | 2.34E-08 | TNRC6C-AS1 |
| ENSG00000182667 | 4.922672824 | -1.199873783 | 3.51E-05 | 0.00014026 | NTM |
| ENSG00000162572 | 33.14783972 | -1.200975478 | 3.31E-06 | 1.55E-05 | SCNN1D |
| ENSG00000137877 | 34.80008523 | -1.205224706 | 9.03E-07 | 4.57E-06 | SPTBN5 |
| ENSG00000198133 | 27.83868881 | -1.206058932 | 5.37E-06 | 2.44E-05 | TMEM229B |
| ENSG00000088340 | 3162.309566 | -1.208315292 | 9.49E-137 | 4.46E-134 | FER1L4 |
| ENSG00000013364 | 3357.820611 | -1.211846974 | 3.09E-140 | 1.58E-137 | MVP |
| ENSG00000189221 | 27.14604725 | -1.214003787 | 7.20E-06 | 3.19E-05 | MAOA |
| ENSG00000245904 | 53.12586267 | -1.214389906 | 7.53E-08 | 4.36E-07 | RP11-796E2.4 |
| ENSG00000166793 | 13.65946745 | -1.216745534 | 6.70E-05 | 0.00025602 | YPEL4 |
| ENSG00000236107 | 37.57020597 | -1.217183681 | 6.34E-07 | 3.28E-06 | AC010127.3 |
| ENSG00000132517 | 13.67876516 | -1.218322593 | 5.68E-05 | 0.00021911 | SLC52A1 |
| ENSG00000164938 | 337.6594351 | -1.218702729 | 4.29E-32 | 1.43E-30 | TP53INP1 |
| ENSG00000161955 | 44.22585449 | -1.219516776 | 1.24E-07 | 7.02E-07 | TNFSF13 |
| ENSG00000102057 | 42.54204618 | -1.220074062 | 1.68E-07 | 9.35E-07 | KCND1 |
| ENSG00000073605 | 548.8614847 | -1.220621342 | 2.15E-52 | 1.74E-50 | GSDMB |
| ENSG00000187210 | 195.431246 | -1.221328875 | 3.9E-20 | 6.46E-19 | GCNT1 |
| ENSG00000174326 | 16.95900453 | -1.222346772 | 3.22E-05 | 0.0001296 | SLC16A11 |
| ENSG00000159403 | 24.22517725 | -1.223671068 | 8.10E-06 | 3.56E-05 | C1R |
| ENSG00000058335 | 60.85782503 | -1.225184652 | 1.31E-09 | 9.24E-09 | RASGRF1 |
| ENSG00000204248 | 61.50431956 | -1.227738248 | 4.76E-09 | 3.17E-08 | COL11A2 |
| ENSG00000158373 | 527.9787573 | -1.228604593 | 1.54E-44 | 9.13E-43 | HIST1H2BD |
| ENSG00000181031 | 82.47177345 | -1.228989084 | 1.51E-11 | 1.28E-10 | RPH3AL |
| ENSG00000182179 | 39.93223649 | -1.229436256 | 2.25E-07 | 1.23E-06 | UBA7 |
| ENSG00000110719 | 541.3408769 | -1.230074854 | 1.69E-48 | 1.2E-46 | TCIRG1 |
| ENSG00000174099 | 25.87089201 | -1.230917655 | 4.42E-06 | 2.03E-05 | MSRB3 |
| ENSG00000105419 | 110.8026117 | -1.231887186 | 3.26E-12 | 2.94E-11 | MEIS3 |
| ENSG00000131037 | 660.3577513 | -1.232305258 | 3.11E-62 | 3.49E-60 | EPS8L1 |
| ENSG00000058404 | 9.540696616 | -1.232875581 | 6.05E-05 | 0.00023265 | CAMK2B |
| ENSG00000187952 | 15.4864835 | -1.234379423 | 4.35E-05 | 0.0001714 | HS6ST1P1 |
| ENSG00000172037 | 1183.878236 | -1.235450403 | 3.07E-91 | 6.77E-89 | LAMB2 |
| ENSG00000230825 | 13.6715639 | -1.235793037 | 4.18E-05 | 0.00016547 | AC005532.5 |
| ENSG00000184730 | 7.88367303 | -1.237274229 | 5.42E-05 | 0.00020984 | APOBR |
| ENSG00000271605 | 37.28981418 | -1.237983181 | 3.33E-07 | 1.78E-06 | MILR1 |
| ENSG00000271795 | 17.58658343 | -1.239251811 | 2.86E-05 | 0.00011612 | CTC-251D13.1 |
| ENSG00000117472 | 4794.352239 | -1.239882574 | 4.1E-174 | 3.01E-171 | TSPAN1 |
| ENSG00000264177 | 43.54489649 | -1.240414863 | 7.55E-08 | 4.37E-07 | RP1-37N7.1 |
| ENSG00000187244 | 1218.753542 | -1.241227218 | 1.75E-87 | 3.61E-85 | BCAM |
| ENSG00000090554 | 9.401560222 | -1.241321375 | 5.38E-05 | 0.00020848 | FLT3LG |
| ENSG00000119457 | 25.05553716 | -1.243870068 | 4.72E-06 | 2.16E-05 | SLC46A2 |
| ENSG00000120327 | 27.20049953 | -1.245652631 | 2.43E-06 | 1.16E-05 | PCDHB14 |
| ENSG00000130518 | 186.751744 | -1.245700215 | 1.3E-19 | 2.09E-18 | KIAA1683 |
| ENSG00000178467 | 164.755684 | -1.250294858 | 6.38E-19 | 9.82E-18 | P4HTM |
| ENSG00000087916 | 80.63138218 | -1.251111111 | 1.22E-11 | 1.05E-10 | SLC6A14 |
| ENSG00000130653 | 27.66600868 | -1.251408261 | 3.02E-06 | 1.43E-05 | PNPLA7 |
| ENSG00000272449 | 23.07406155 | -1.251490951 | 7.12E-06 | 3.16E-05 | RP3-395M20.12 |
| ENSG00000204176 | 278.1554663 | -1.253008372 | 2.23E-29 | 6.28E-28 | SYT15 |
| ENSG00000177076 | 246.285009 | -1.253633821 | 1.97E-25 | 4.52E-24 | ACER2 |
| ENSG00000156042 | 24.54062682 | -1.254797599 | 3.86E-06 | 1.79E-05 | TTC18 |
| ENSG00000165125 | 36.60279604 | -1.258176557 | 1.13E-06 | 5.66E-06 | TRPV6 |
| ENSG00000228486 | 18.95045345 | -1.25962708 | 1.16E-05 | 4.98E-05 | LINC01125 |
| ENSG00000129270 | 1301.446612 | -1.260828862 | 1.09E-98 | 2.73E-96 | MMP28 |
| ENSG00000235863 | 60.26376231 | -1.264217759 | 4.92E-09 | 3.27E-08 | B3GALT4 |
| ENSG00000128833 | 322.8877388 | -1.267046826 | 1.38E-34 | 5.2E-33 | MYO5C |
| ENSG00000184343 | 42.52022312 | -1.267465279 | 5.52E-08 | 3.25E-07 | SRPK3 |
| ENSG00000176809 | 88.57922007 | -1.269276261 | 1.74E-11 | 1.46E-10 | LRRC37A3 |
| ENSG00000183111 | 435.8192555 | -1.269883953 | 9.43E-45 | 5.65E-43 | ARHGEF37 |
| ENSG00000183778 | 21.58418891 | -1.273258766 | 6.30E-06 | 2.83E-05 | B3GALT5 |
| ENSG00000127329 | 216.1009504 | -1.273440784 | 1.7E-25 | 3.92E-24 | PTPRB |
| ENSG00000273336 | 22.09151573 | -1.278402643 | 7.38E-06 | 3.27E-05 | OR7M1P |
| ENSG00000231187 | 103.7472351 | -1.278676946 | 3.46E-14 | 3.76E-13 | RP11-38L15.3 |
| ENSG00000053438 | 37.42143418 | -1.278871254 | 2.54E-07 | 1.38E-06 | NNAT |
| ENSG00000130487 | 7.71185003 | -1.279600385 | 2.84E-05 | 0.00011545 | KLHDC7B |
| ENSG00000130234 | 14.1377388 | -1.281174087 | 2.07E-05 | 8.57E-05 | ACE2 |
| ENSG00000232316 | 8.870860762 | -1.287641756 | 2.80E-05 | 0.00011386 | RP1-124C6.1 |
| ENSG00000254548 | 36.3748963 | -1.293218454 | 3.93E-07 | 2.09E-06 | RP11-429J17.5 |
| ENSG00000179698 | 38.21519775 | -1.295532623 | 2.69E-07 | 1.45E-06 | KIAA1875 |
| ENSG00000226674 | 5.414662842 | -1.295754032 | 1.06E-05 | 4.60E-05 | TEX41 |
| ENSG00000265150 | 2989.495994 | -1.297085058 | 3.58E-09 | 2.42E-08 | RN7SL2 |
| ENSG00000172232 | 31.04859571 | -1.297408206 | 1.55E-06 | 7.61E-06 | AZU1 |
| ENSG00000086570 | 1258.628152 | -1.299745446 | 1.67E-25 | 3.85E-24 | FAT2 |
| ENSG00000205890 | 195.6543268 | -1.29980533 | 4.49E-25 | 1E-23 | RP11-473M20.5 |
| ENSG00000168453 | 455.1722172 | -1.302003322 | 1.6E-48 | 1.14E-46 | HR |
| ENSG00000158186 | 19.43162848 | -1.302067537 | 5.22E-06 | 2.37E-05 | MRAS |
| ENSG00000223764 | 153.8629001 | -1.302553451 | 7.64E-20 | 1.24E-18 | RP11-54O7.3 |
| ENSG00000262884 | 32.99658724 | -1.302601126 | 2.76E-07 | 1.49E-06 | CTD-3060P21.1 |
| ENSG00000214279 | 463.9067377 | -1.304598914 | 2.15E-42 | 1.16E-40 | RP11-108K14.4 |
| ENSG00000064687 | 965.1232986 | -1.304726763 | 3.52E-82 | 6.27E-80 | ABCA7 |
| ENSG00000167995 | 27.68525431 | -1.309834903 | 6.71E-07 | 3.46E-06 | BEST1 |
| ENSG00000112769 | 15.99391698 | -1.310022123 | 1.20E-05 | 5.14E-05 | LAMA4 |
| ENSG00000138764 | 496.7677496 | -1.311562568 | 6.31E-54 | 5.31E-52 | CCNG2 |
| ENSG00000149043 | 184.494091 | -1.311963133 | 3.87E-21 | 6.78E-20 | SYT8 |
| ENSG00000106541 | 2628.640606 | -1.312741012 | 2.21E-123 | 8.45E-121 | AGR2 |
| ENSG00000114854 | 49.92927997 | -1.312798784 | 2.33E-09 | 1.61E-08 | TNNC1 |
| ENSG00000265298 | 112.9518378 | -1.314930842 | 7.33E-13 | 7.07E-12 | RP13-104F24.3 |
| ENSG00000162931 | 66.64796354 | -1.318423598 | 4.31E-11 | 3.5E-10 | TRIM17 |
| ENSG00000171124 | 882.5324088 | -1.319020135 | 5.46E-78 | 8.7E-76 | FUT3 |
| ENSG00000167608 | 1499.292355 | -1.320515755 | 3.04E-129 | 1.27E-126 | TMC4 |
| ENSG00000090006 | 52.18018891 | -1.320894325 | 1.66E-08 | 1.04E-07 | LTBP4 |
| ENSG00000163297 | 82.83779482 | -1.322389279 | 7.58E-12 | 6.61E-11 | ANTXR2 |
| ENSG00000198569 | 94.08260685 | -1.322536286 | 8.1E-13 | 7.78E-12 | SLC34A3 |
| ENSG00000197822 | 1353.067441 | -1.322783149 | 1.58E-85 | 3.14E-83 | OCLN |
| ENSG00000106976 | 45.48344026 | -1.325409 | 4.27E-09 | 2.86E-08 | DNM1 |
| ENSG00000183508 | 41.32293139 | -1.327072153 | 7.99E-08 | 4.61E-07 | FAM46C |
| ENSG00000244541 | 12.67453843 | -1.329333793 | 1.17E-05 | 5.02E-05 | RP11-167H9.6 |
| ENSG00000196966 | 35.40470115 | -1.329763036 | 1.05E-07 | 5.98E-07 | HIST1H3E |
| ENSG00000253910 | 12.67146463 | -1.33062412 | 1.14E-05 | 4.91E-05 | PCDHGB2 |
| ENSG00000019169 | 19.41737665 | -1.330792562 | 3.81E-06 | 1.77E-05 | MARCO |
| ENSG00000012171 | 84.10063394 | -1.331202175 | 2.37E-13 | 2.38E-12 | SEMA3B |
| ENSG00000100234 | 550.7876763 | -1.331426019 | 7.4E-59 | 7.18E-57 | TIMP3 |
| ENSG00000124006 | 333.325676 | -1.334727593 | 2.26E-39 | 1.08E-37 | OBSL1 |
| ENSG00000269640 | 12.67954073 | -1.335316861 | 1.24E-05 | 5.30E-05 | CTD-2521M24.9 |
| ENSG00000133328 | 40.7093564 | -1.336548129 | 1.84E-08 | 1.14E-07 | HRASLS2 |
| ENSG00000197142 | 1299.031758 | -1.337478059 | 3.89E-108 | 1.23E-105 | ACSL5 |
| ENSG00000136542 | 146.5040267 | -1.33764757 | 2.12E-20 | 3.57E-19 | GALNT5 |
| ENSG00000129595 | 68.43545858 | -1.338511073 | 9.79E-12 | 8.46E-11 | EPB41L4A |
| ENSG00000167653 | 609.2861731 | -1.3396204 | 5.48E-66 | 7.02E-64 | PSCA |
| ENSG00000119922 | 907.1576088 | -1.339895183 | 4.44E-86 | 8.93E-84 | IFIT2 |
| ENSG00000229807 | 7.755859374 | -1.340101039 | 8.96E-06 | 3.91E-05 | XIST |
| ENSG00000235703 | 121.1343108 | -1.340667182 | 1.61E-16 | 2.08E-15 | LINC00894 |
| ENSG00000119943 | 237.0969553 | -1.342337192 | 1.68E-29 | 4.78E-28 | PYROXD2 |
| ENSG00000072195 | 35.59406737 | -1.343341056 | 6.58E-08 | 3.84E-07 | SPEG |
| ENSG00000171401 | 3585.087563 | -1.34496229 | 2.24E-174 | 1.71E-171 | KRT13 |
| ENSG00000129009 | 26.16051825 | -1.345783451 | 6.65E-07 | 3.43E-06 | ISLR |
| ENSG00000102886 | 139.5301315 | -1.345963763 | 2.16E-19 | 3.41E-18 | GDPD3 |
| ENSG00000243566 | 1088.945419 | -1.346349712 | 1.61E-82 | 2.95E-80 | UPK3B |
| ENSG00000185885 | 61.13698915 | -1.347869809 | 6.58E-11 | 5.27E-10 | IFITM1 |
| ENSG00000179023 | 6.412859385 | -1.348729762 | 7.03E-06 | 3.13E-05 | KLHDC7A |
| ENSG00000183035 | 38.22633156 | -1.350917212 | 3.41E-08 | 2.06E-07 | CYLC1 |
| ENSG00000187837 | 369.3987191 | -1.358088363 | 2.84E-36 | 1.16E-34 | HIST1H1C |
| ENSG00000197536 | 89.52656749 | -1.360564527 | 1.21E-14 | 1.37E-13 | C5orf56 |
| ENSG00000213901 | 56.55345151 | -1.366488265 | 6.68E-10 | 4.85E-09 | SLC23A3 |
| ENSG00000063180 | 42.03980924 | -1.36711786 | 6.4E-09 | 4.19E-08 | CA11 |
| ENSG00000186007 | 141.3274035 | -1.369042376 | 1.28E-18 | 1.92E-17 | LEMD1 |
| ENSG00000163462 | 23.89704272 | -1.369706592 | 5.61E-07 | 2.92E-06 | TRIM46 |
| ENSG00000112038 | 21.74078427 | -1.376477988 | 9.91E-07 | 4.98E-06 | OPRM1 |
| ENSG00000248092 | 7.212297058 | -1.377979409 | 5.68E-06 | 2.56E-05 | NNT-AS1 |
| ENSG00000175318 | 42.20114973 | -1.380931485 | 3.12E-09 | 2.13E-08 | GRAMD2 |
| ENSG00000105388 | 385.3326545 | -1.382273872 | 1.17E-45 | 7.33E-44 | CEACAM5 |
| ENSG00000100100 | 270.499189 | -1.383474521 | 8.59E-35 | 3.29E-33 | PIK3IP1 |
| ENSG00000006534 | 1380.539563 | -1.384158305 | 3.4E-108 | 1.09E-105 | ALDH3B1 |
| ENSG00000236830 | 43.51443314 | -1.387859994 | 5.38E-09 | 3.56E-08 | CBR3-AS1 |
| ENSG00000116701 | 139.1174818 | -1.394634648 | 1.11E-18 | 1.67E-17 | NCF2 |
| ENSG00000115919 | 37.37081615 | -1.396948112 | 3.24E-08 | 1.96E-07 | KYNU |
| ENSG00000260193 | 32.76760601 | -1.398793849 | 5.96E-08 | 3.50E-07 | RP11-83N9.5 |
| ENSG00000140675 | 20.08655348 | -1.399824011 | 8.69E-07 | 4.41E-06 | SLC5A2 |
| ENSG00000173567 | 34.93541874 | -1.402859666 | 1.87E-08 | 1.16E-07 | GPR113 |
| ENSG00000224940 | 31.16497362 | -1.404064488 | 9.34E-08 | 5.37E-07 | PRRT4 |
| ENSG00000166387 | 347.9034319 | -1.406206181 | 4.83E-43 | 2.69E-41 | PPFIBP2 |
| ENSG00000172159 | 25.16827983 | -1.411021075 | 6.21E-07 | 3.21E-06 | FRMD3 |
| ENSG00000196337 | 53.23235311 | -1.414261916 | 5.31E-10 | 3.9E-09 | CGB7 |
| ENSG00000172824 | 94.63078031 | -1.415927137 | 1.92E-16 | 2.47E-15 | CES4A |
| ENSG00000078814 | 93.15161467 | -1.425123098 | 9.67E-14 | 1.01E-12 | MYH7B |
| ENSG00000047346 | 409.0537311 | -1.425140271 | 3.34E-52 | 2.68E-50 | FAM214A |
| ENSG00000020181 | 18.78459254 | -1.425447934 | 8.91E-07 | 4.51E-06 | GPR124 |
| ENSG00000177694 | 82.47394013 | -1.425621091 | 1.46E-14 | 1.63E-13 | NAALADL2 |
| ENSG00000188897 | 39.37636042 | -1.43351612 | 8.2E-09 | 5.31E-08 | CTD-3088G3.8 |
| ENSG00000177409 | 480.6620743 | -1.43581715 | 6.86E-47 | 4.62E-45 | SAMD9L |
| ENSG00000106560 | 7.560727413 | -1.437666731 | 2.39E-06 | 1.14E-05 | GIMAP2 |
| ENSG00000174963 | 69.22051201 | -1.443889263 | 9.03E-13 | 8.62E-12 | ZIC4 |
| ENSG00000089356 | 3283.347923 | -1.445457109 | 1.86E-23 | 3.72E-22 | FXYD3 |
| ENSG00000159450 | 59.62450119 | -1.44772576 | 4.58E-12 | 4.06E-11 | TCHH |
| ENSG00000174501 | 401.0546318 | -1.452019305 | 2.07E-38 | 9.41E-37 | ANKRD36C |
| ENSG00000241769 | 48.96473532 | -1.454727793 | 2.11E-10 | 1.61E-09 | LINC00893 |
| ENSG00000260765 | 10.66030178 | -1.458830956 | 2.08E-06 | 1.00E-05 | CES1P2 |
| ENSG00000168016 | 167.8466543 | -1.459542075 | 3.47E-25 | 7.84E-24 | TRANK1 |
| ENSG00000182612 | 43.69279375 | -1.459955092 | 3.72E-10 | 2.76E-09 | TSPAN10 |
| ENSG00000136999 | 17.95522664 | -1.461875569 | 5.70E-07 | 2.97E-06 | NOV |
| ENSG00000116852 | 52.15081417 | -1.462953948 | 1.19E-09 | 8.46E-09 | KIF21B |
| ENSG00000186529 | 229.3787156 | -1.465319282 | 1.74E-33 | 6.21E-32 | CYP4F3 |
| ENSG00000141574 | 466.3277669 | -1.470994328 | 1.42E-57 | 1.32E-55 | SECTM1 |
| ENSG00000140297 | 121.0470772 | -1.472855644 | 1.93E-17 | 2.65E-16 | GCNT3 |
| ENSG00000106789 | 1382.668287 | -1.475255225 | 2.47E-138 | 1.22E-135 | CORO2A |
| ENSG00000169604 | 658.9802778 | -1.475360152 | 4.89E-79 | 8.07E-77 | ANTXR1 |
| ENSG00000160111 | 13.66994247 | -1.476976587 | 1.07E-06 | 5.34E-06 | CPAMD8 |
| ENSG00000125775 | 237.2316587 | -1.47779565 | 1.66E-32 | 5.63E-31 | SDCBP2 |
| ENSG00000166750 | 1599.657294 | -1.480990528 | 2.1E-150 | 1.24E-147 | SLFN5 |
| ENSG00000170298 | 31.28678098 | -1.482312812 | 1.05E-07 | 5.98E-07 | LGALS9B |
| ENSG00000165025 | 37.21848249 | -1.484642124 | 3.43E-09 | 2.33E-08 | SYK |
| ENSG00000176485 | 1653.435486 | -1.484676782 | 2.31E-169 | 1.51E-166 | PLA2G16 |
| ENSG00000155324 | 365.6092205 | -1.484741268 | 2.93E-49 | 2.11E-47 | GRAMD3 |
| ENSG00000223573 | 331.2918383 | -1.487024897 | 4.71E-47 | 3.21E-45 | TINCR |
| ENSG00000205220 | 298.0830669 | -1.487039318 | 2.16E-43 | 1.22E-41 | PSMB10 |
| ENSG00000100156 | 66.16755758 | -1.488991543 | 1.07E-12 | 1.01E-11 | SLC16A8 |
| ENSG00000173267 | 1919.331251 | -1.490179398 | 4.39E-39 | 2.05E-37 | SNCG |
| ENSG00000162576 | 275.99186 | -1.490933019 | 8.56E-40 | 4.15E-38 | MXRA8 |
| ENSG00000164626 | 122.2896491 | -1.494203558 | 2.25E-19 | 3.54E-18 | KCNK5 |
| ENSG00000196739 | 50.45449866 | -1.494758327 | 2.26E-11 | 1.88E-10 | COL27A1 |
| ENSG00000260912 | 123.1490779 | -1.497253458 | 1.88E-19 | 2.98E-18 | RP11-363E7.4 |
| ENSG00000108602 | 622.9651701 | -1.498526474 | 1.3E-81 | 2.29E-79 | ALDH3A1 |
| ENSG00000082014 | 47.82376796 | -1.502686638 | 8.57E-11 | 6.8E-10 | SMARCD3 |
| ENSG00000137501 | 1636.542862 | -1.503020577 | 2.05E-149 | 1.17E-146 | SYTL2 |
| ENSG00000129451 | 20.21769795 | -1.505297571 | 2.13E-07 | 1.17E-06 | KLK10 |
| ENSG00000146021 | 157.2235389 | -1.509845614 | 2.23E-26 | 5.42E-25 | KLHL3 |
| ENSG00000130303 | 38.50046678 | -1.509942205 | 1.5E-09 | 1.06E-08 | BST2 |
| ENSG00000127084 | 553.0664964 | -1.511040361 | 2.24E-73 | 3.26E-71 | FGD3 |
| ENSG00000269516 | 121.4100629 | -1.512302448 | 8.26E-20 | 1.34E-18 | CYP4F23P |
| ENSG00000186765 | 22.91406305 | -1.516748127 | 8.19E-08 | 4.73E-07 | FSCN2 |
| ENSG00000188833 | 94.69066122 | -1.517812021 | 1.48E-15 | 1.78E-14 | ENTPD8 |
| ENSG00000114631 | 24.66797233 | -1.52034395 | 7.13E-08 | 4.14E-07 | PODXL2 |
| ENSG00000110375 | 71.81957502 | -1.520820086 | 6.86E-15 | 7.87E-14 | UPK2 |
| ENSG00000261068 | 211.6732666 | -1.528589992 | 2.01E-31 | 6.43E-30 | RP11-7K24.3 |
| ENSG00000163283 | 101.5777081 | -1.533815322 | 1.67E-18 | 2.48E-17 | ALPP |
| ENSG00000124249 | 17.90363248 | -1.538943485 | 1.84E-07 | 1.02E-06 | KCNK15 |
| ENSG00000198467 | 60.78336004 | -1.539055792 | 1.41E-13 | 1.46E-12 | TPM2 |
| ENSG00000089012 | 7.704366464 | -1.541310547 | 4.11E-07 | 2.17E-06 | SIRPG |
| ENSG00000218416 | 161.0712238 | -1.546988082 | 5.11E-28 | 1.35E-26 | AC110619.2 |
| ENSG00000225548 | 18.93024394 | -1.54754607 | 9.16E-08 | 5.28E-07 | AC098973.2 |
| ENSG00000130707 | 657.7998711 | -1.551183556 | 5.47E-94 | 1.28E-91 | ASS1 |
| ENSG00000136689 | 571.3856055 | -1.559227649 | 1.55E-83 | 2.87E-81 | IL1RN |
| ENSG00000116039 | 15.78857241 | -1.559807412 | 1.67E-07 | 9.31E-07 | ATP6V1B1 |
| ENSG00000142973 | 20.40290025 | -1.562586915 | 4.42E-08 | 2.63E-07 | CYP4B1 |
| ENSG00000117289 | 5793.605278 | -1.565872823 | 6.92E-296 | 2.11E-292 | TXNIP |
| ENSG00000012822 | 816.8837192 | -1.567058307 | 1.92E-111 | 6.38E-109 | CALCOCO1 |
| ENSG00000108064 | 776.9624059 | -1.570537342 | 1.14E-88 | 2.39E-86 | TFAM |
| ENSG00000206538 | 199.5734941 | -1.577629108 | 2.42E-35 | 9.51E-34 | VGLL3 |
| ENSG00000135363 | 50.37442637 | -1.579934426 | 2.34E-12 | 2.13E-11 | LMO2 |
| ENSG00000168477 | 277.7035647 | -1.580125572 | 6.41E-42 | 3.39E-40 | TNXB |
| ENSG00000112902 | 102.9784028 | -1.58627941 | 4.04E-20 | 6.69E-19 | SEMA5A |
| ENSG00000273032 | 33.1149128 | -1.588857108 | 1.51E-09 | 1.06E-08 | DGCR9 |
| ENSG00000214860 | 80.43192965 | -1.590684542 | 8.47E-17 | 1.11E-15 | EVPLL |
| ENSG00000181085 | 394.9526776 | -1.591177935 | 4.3E-58 | 4.08E-56 | MAPK15 |
| ENSG00000168918 | 135.0344128 | -1.591575468 | 9.26E-25 | 2.03E-23 | INPP5D |
| ENSG00000162645 | 139.881014 | -1.597301588 | 3.38E-26 | 8.16E-25 | GBP2 |
| ENSG00000171236 | 139.1263069 | -1.601401489 | 1.36E-25 | 3.16E-24 | LRG1 |
| ENSG00000079385 | 633.586538 | -1.603954393 | 1.2E-90 | 2.62E-88 | CEACAM1 |
| ENSG00000211448 | 16.25947444 | -1.604524199 | 9.44E-08 | 5.43E-07 | DIO2 |
| ENSG00000164342 | 243.7631263 | -1.608072484 | 2.37E-42 | 1.27E-40 | TLR3 |
| ENSG00000165895 | 28.95084966 | -1.610429507 | 1.79E-09 | 1.25E-08 | ARHGAP42 |
| ENSG00000105357 | 2028.121513 | -1.618261216 | 2.98E-179 | 2.48E-176 | MYH14 |
| ENSG00000163083 | 17.0829662 | -1.625256755 | 5.15E-08 | 3.05E-07 | INHBB |
| ENSG00000184584 | 1812.414813 | -1.626427166 | 3.19E-196 | 3.08E-193 | TMEM173 |
| ENSG00000175155 | 159.1723884 | -1.62764896 | 1.44E-28 | 3.91E-27 | YPEL2 |
| ENSG00000003400 | 42.97488431 | -1.629661135 | 8.3E-12 | 7.22E-11 | CASP10 |
| ENSG00000265787 | 17.13231109 | -1.63042705 | 4.57E-08 | 2.72E-07 | CYP4F35P |
| ENSG00000153233 | 607.5039567 | -1.635664343 | 3.51E-82 | 6.27E-80 | PTPRR |
| ENSG00000169583 | 532.829374 | -1.643472243 | 4.44E-69 | 6.26E-67 | CLIC3 |
| ENSG00000196872 | 168.2513604 | -1.659484687 | 3.3E-33 | 1.15E-31 | KIAA1211L |
| ENSG00000145217 | 48.10922652 | -1.672151008 | 2.46E-13 | 2.47E-12 | SLC26A1 |
| ENSG00000116106 | 195.8410519 | -1.676251891 | 1.1E-35 | 4.39E-34 | EPHA4 |
| ENSG00000268287 | 18.08103351 | -1.676726433 | 1.26E-08 | 7.94E-08 | CTB-60B18.18 |
| ENSG00000172915 | 104.1259933 | -1.6791426 | 1.45E-22 | 2.76E-21 | NBEA |
| ENSG00000163131 | 62.54074639 | -1.683662837 | 4.26E-15 | 4.98E-14 | CTSS |
| ENSG00000073737 | 88.622743 | -1.696301933 | 2.06E-19 | 3.25E-18 | DHRS9 |
| ENSG00000197496 | 153.4485776 | -1.706482908 | 4.28E-28 | 1.14E-26 | SLC2A10 |
| ENSG00000255735 | 52.05293805 | -1.707856579 | 1.98E-14 | 2.19E-13 | AC110619.1 |
| ENSG00000170835 | 127.4576053 | -1.71714633 | 2.85E-25 | 6.48E-24 | CEL |
| ENSG00000169432 | 33.74501716 | -1.725056274 | 1.56E-11 | 1.32E-10 | SCN9A |
| ENSG00000139626 | 70.79813534 | -1.725171675 | 9.57E-17 | 1.25E-15 | ITGB7 |
| ENSG00000124467 | 164.3291724 | -1.726775757 | 9.06E-33 | 3.1E-31 | PSG8 |
| ENSG00000173269 | 353.4411625 | -1.73169497 | 5.31E-65 | 6.62E-63 | MMRN2 |
| ENSG00000112782 | 35.00842009 | -1.755526019 | 3.28E-11 | 2.69E-10 | CLIC5 |
| ENSG00000163141 | 122.9949928 | -1.762333716 | 5.94E-28 | 1.56E-26 | BNIPL |
| ENSG00000108244 | 202.40686 | -1.773278451 | 3.22E-39 | 1.53E-37 | KRT23 |
| ENSG00000149596 | 88.41168396 | -1.807109405 | 1.1E-22 | 2.11E-21 | JPH2 |
| ENSG00000135373 | 855.7253639 | -1.814713425 | 2.67E-143 | 1.49E-140 | EHF |
| ENSG00000108947 | 63.05430365 | -1.816348981 | 4.44E-18 | 6.43E-17 | EFNB3 |
| ENSG00000131398 | 205.0960433 | -1.816402441 | 2.47E-41 | 1.27E-39 | KCNC3 |
| ENSG00000230606 | 117.0758941 | -1.819936973 | 1.23E-28 | 3.36E-27 | AC159540.1 |
| ENSG00000170017 | 624.3030125 | -1.830932084 | 4.2E-110 | 1.37E-107 | ALCAM |
| ENSG00000244242 | 227.9573056 | -1.830973754 | 2.07E-46 | 1.35E-44 | IFITM10 |
| ENSG00000143365 | 23.1512161 | -1.83558346 | 1.75E-10 | 1.34E-09 | RORC |
| ENSG00000143653 | 146.0457931 | -1.848335697 | 6.88E-35 | 2.64E-33 | SCCPDH |
| ENSG00000198157 | 9.860714659 | -1.880474534 | 8.69E-10 | 6.25E-09 | HMGN5 |
| ENSG00000137752 | 62.57219306 | -1.892237137 | 1.83E-18 | 2.71E-17 | CASP1 |
| ENSG00000258486 | 2426.393611 | -1.910365859 | 5.89E-15 | 6.78E-14 | RN7SL1 |
| ENSG00000146674 | 6021.533442 | -1.922707847 | 0 | 0 | IGFBP3 |
| ENSG00000094755 | 186.1031855 | -1.930757057 | 5.44E-43 | 3.01E-41 | GABRP |
| ENSG00000110042 | 430.0838798 | -1.953527176 | 6.2E-92 | 1.39E-89 | DTX4 |
| ENSG00000167741 | 136.5127264 | -1.956769875 | 3.26E-36 | 1.32E-34 | GGT6 |
| ENSG00000025423 | 19.5759718 | -1.95976799 | 2.59E-11 | 2.15E-10 | HSD17B6 |
| ENSG00000186204 | 662.5827433 | -1.965379882 | 1.76E-130 | 7.85E-128 | CYP4F12 |
| ENSG00000166920 | 208.9411636 | -1.972831383 | 7.4E-46 | 4.68E-44 | C15orf48 |
| ENSG00000137975 | 49.16841648 | -1.975873537 | 4.63E-17 | 6.18E-16 | CLCA2 |
| ENSG00000124593 | 282.3113844 | -1.990711621 | 7.39E-62 | 8.11E-60 | PRICKLE4 |
| ENSG00000232931 | 576.2143155 | -2.000659104 | 5.03E-126 | 2E-123 | LINC00342 |
| ENSG00000167183 | 158.9005512 | -2.049570594 | 1.64E-43 | 9.3E-42 | PRR15L |
| ENSG00000198520 | 101.9463159 | -2.052987262 | 2.09E-29 | 5.89E-28 | C1orf228 |
| ENSG00000178826 | 113.8898057 | -2.053080536 | 2.2E-32 | 7.43E-31 | TMEM139 |
| ENSG00000137648 | 407.0060048 | -2.068929329 | 2.07E-75 | 3.17E-73 | TMPRSS4 |
| ENSG00000159921 | 475.8123497 | -2.115822935 | 1.4E-107 | 4.28E-105 | GNE |
| ENSG00000168961 | 131.0709046 | -2.116791995 | 2.7E-37 | 1.17E-35 | LGALS9 |
| ENSG00000111644 | 20.87703834 | -2.120426655 | 3.83E-13 | 3.78E-12 | ACRBP |
| ENSG00000145113 | 119.5107406 | -2.126075697 | 4.26E-36 | 1.72E-34 | MUC4 |
| ENSG00000064787 | 186.3638577 | -2.13320464 | 6.67E-51 | 5.18E-49 | BCAS1 |
| ENSG00000271880 | 18.86481327 | -2.144632248 | 6.59E-13 | 6.38E-12 | RP11-96C23.5 |
| ENSG00000153902 | 38.12127584 | -2.181319461 | 2.53E-17 | 3.45E-16 | LGI4 |
| ENSG00000102287 | 525.1119228 | -2.2305826 | 4.13E-138 | 1.99E-135 | GABRE |
| ENSG00000035664 | 106.4724767 | -2.23821487 | 4.69E-36 | 1.89E-34 | DAPK2 |
| ENSG00000132139 | 28.61697709 | -2.264654486 | 4.99E-16 | 6.22E-15 | GAS2L2 |
| ENSG00000167754 | 178.2935108 | -2.280976336 | 2.52E-55 | 2.21E-53 | KLK5 |
| ENSG00000125735 | 13.77618459 | -2.292416425 | 8.1E-14 | 8.56E-13 | TNFSF14 |
| ENSG00000224769 | 218.9811594 | -2.301685816 | 1.01E-52 | 8.21E-51 | AC069213.1 |
| ENSG00000091592 | 81.92961329 | -2.30286377 | 1.07E-30 | 3.27E-29 | NLRP1 |
| ENSG00000131711 | 72.72315426 | -2.309124827 | 2.94E-25 | 6.66E-24 | MAP1B |
| ENSG00000106078 | 82.5838553 | -2.31842527 | 1.92E-31 | 6.15E-30 | COBL |
| ENSG00000134258 | 288.6108093 | -2.331872756 | 1.34E-83 | 2.5E-81 | VTCN1 |
| ENSG00000132274 | 77.96235042 | -2.353042556 | 2.69E-28 | 7.21E-27 | TRIM22 |
| ENSG00000174080 | 52.93974525 | -2.375696795 | 1.71E-22 | 3.24E-21 | CTSF |
| ENSG00000145864 | 16.76534901 | -2.458066459 | 9.63E-16 | 1.18E-14 | GABRB2 |
| ENSG00000136059 | 31.0883398 | -2.470560845 | 8.46E-19 | 1.29E-17 | VILL |
| ENSG00000184985 | 189.8074107 | -2.484738965 | 4.41E-61 | 4.67E-59 | SORCS2 |
| ENSG00000163993 | 663.7427619 | -2.509613366 | 6.4E-162 | 4.05E-159 | S100P |
| ENSG00000176945 | 1663.661601 | -2.783644587 | 0 | 0 | MUC20 |
| ENSG00000179776 | 267.4411211 | -2.822488155 | 1.87E-101 | 4.89E-99 | CDH5 |
| ENSG00000181143 | 4810.632969 | -2.836994107 | 3.98E-59 | 3.94E-57 | MUC16 |
| ENSG00000133321 | 142.2562642 | -2.957352906 | 2.42E-60 | 2.49E-58 | RARRES3 |
| ENSG00000162981 | 972.9939371 | -2.989705771 | 0 | 0 | FAM84A |
| ENSG00000148346 | 219.6100416 | -3.061563309 | 4.97E-94 | 1.18E-91 | LCN2 |
| ENSG00000124107 | 187.9772919 | -3.103084023 | 8.89E-81 | 1.55E-78 | SLPI |
| ENSG00000102390 | 49.25825081 | -3.29079214 | 3.72E-35 | 1.45E-33 | PBDC1 |
| ENSG00000112992 | 32.64460503 | -3.371108557 | 1E-30 | 3.1E-29 | NNT |
| ENSG00000125730 | 942.7486245 | -3.499436932 | 0 | 0 | C3 |
| ENSG00000131171 | 243.986697 | -5.49837947 | 8.92E-124 | 3.48E-121 | SH3BGRL |
